# Hetero-Solvent Microenvironment for Selective CO_2_ to Ethanol Electrolysis *via* Interfacial Water Control

**DOI:** 10.1007/s40820-026-02282-w

**Published:** 2026-07-03

**Authors:** Dohun Kim, Suyun Lee, Seeun Jung, Jaemin Kim, Junsic Cho, Dong Ki Lee, Seoin Back, Chang Hyuck Choi, Chanyeon Kim

**Affiliations:** 1 Department of Energy Science and Engineering, Daegu Gyeongbuk Institute of Science and Technology (DGIST), Daegu, 42988 Republic of Korea; 2 Department of Chemistry, Pohang University of Science and Technology (POSTECH), Pohang, 37673 Republic of Korea; 3 Department of Chemical and Biomolecular Engineering, Sogang University, Seoul, 04107 Republic of Korea; 4 Clean Energy Research Center, Korea Institute of Science and Technology, Seoul, 02792 Republic of Korea; 5 Department of Chemical and Biomolecular Engineering, Yonsei-KIST Convergence Research Institute, Yonsei University, Seoul, 03722 Republic of Korea; 6 Department of Integrative Energy Engineering, Korea University, Seoul, 02841 Republic of Korea; 7 KU-KIST Graduate School of Converging Science and Technology, Korea University, 145 Anam-ro, Seongbuk-gu, Seoul, 02841 Republic of Korea; 8 Institute for Multiscale Matter and Systems (IMMS), Ewha Womans University, Seoul, 03760 Republic of Korea

**Keywords:** Electrochemical CO_2_ reduction, Microenvironment, Diglyme, Hetero-solvent, Ethanol production

## Abstract

Electrochemical reduction of carbon dioxide (CO_2_RR) offers a route for sustainable chemical production using water as a clean proton source. However, water also promotes the competing hydrogen evolution reaction, limiting CO_2_RR performance. Here we establish interfacial water as a decisive but overlooked design parameter for selective CO_2_-to-ethanol electrolysis. A hetero-solvent microenvironment confining diglyme (DiG) near the Cu catalyst substantially suppresses HER under both neutral and alkaline conditions, where protons are supplied *via* water dissociation. *In situ* infrared absorption spectroscopy and theoretical calculation results reveal that DiG strengthens the hydrogen-bonding network of interfacial water, reducing free-water population prone to dissociation. Concurrently, the modulated water network effectively hinders solvent-mediated hydrogenation that favors ethylene formation, thereby promoting ethanol formation. Because this strategy modulates the microenvironment rather than the catalyst, it readily extends to Cu–Ag bimetallic catalyst. Moreover, confining hetero-solvent within microenvironment rather than in the bulk electrolyte enables high-current operation at low cell voltages, achieving an ethanol partial current density of 184.2 mA cm^−2^ at 3.6 V under neutral condition.

**Supplementary Information:**

The online version contains supplementary material available at 10.1007/s40820-026-02282-w.

## Highlights

- A hetero-solvent-confined microenvironment regulates interfacial water during CO_2_ electrolysis on Cu, substantially suppressing hydrogen evolution by inhibiting water dissociation.
- Inhibited water dissociation suppresses ethylene formation, which requires solvent-mediated hydrogenation, thereby steering selectivity toward ethanol.
- This strategy is readily extended to Cu–Ag catalyst, enabling record-high ethanol production under neutral conditions while minimizing cell voltage by confining hetero-solvent within microenvironment rather than bulk electrolyte.

**Supplementary Information:**

The online version contains supplementary material available at 10.1007/s40820-026-02282-w.

## Graphical abstract

**Figure:**
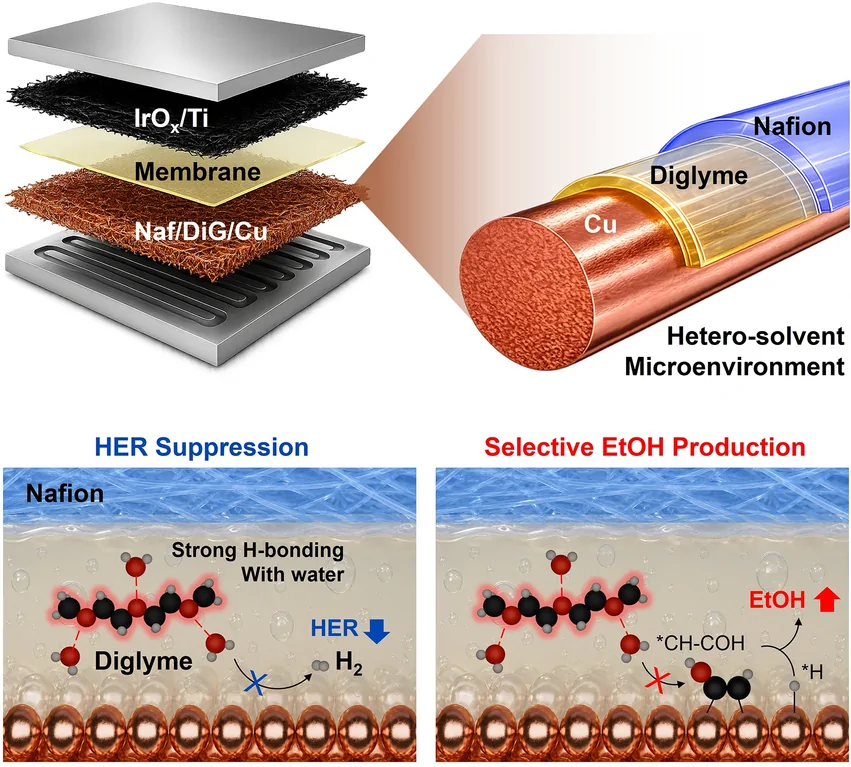

## Introduction

The electrochemical CO_2_ reduction reaction (CO_2_RR) enables the conversion of CO_2_ with water into value-added chemicals and fuels. Since CO_2_RR uses water as the proton source, it holds great potential for sustainable chemical and fuel production when it is integrated with renewably generated electricity [1–3]. However, the use of water also limits the activity and selectivity toward CO_2_RR. In aqueous electrolytes, a large disparity between water concentration (~ 55.6 M) and dissolved CO_2_ concentration (~ 34 mM) results in a low local CO_2_/H_2_O ratio near the catalyst surface, which in turn favors the hydrogen evolution reaction (HER) over CO_2_RR [4, 5].

To mitigate competing HER, previous studies have focused on tuning the hydrophobicity of the catalyst surface to inhibit the access of water [6–9]. However, excessive hydrophobicity can also impede CO_2_RR activity by limiting the number of active sites in contact with electrolyte for proton transfer [6, 10, 11]. Therefore, controlling hydrophobicity alone cannot fully resolve issues on CO_2_RR activity and selectivity caused by HER. Another approach to suppress HER involves modifying the pH of the aqueous electrolytes. Numerous studies have reported HER suppression by increasing pH in either the bulk electrolyte or the catalytic microenvironment [4, 10, 12–14]. However, even under high pH such as neutral and alkaline conditions, HER can still occur *via* the Volmer step involving water dissociation (H_2_O + e^−^ → H* + OH^−^) [15, 16]. Therefore, further inhibition of HER requires control over water dissociation.

The HER activity is closely associated with the hydrogen-bonding network of interfacial water molecules. When HER is promoted, an increased population of free-water, or less hydrogen-bonded structure, is observed at the interface, suggesting that a higher free-water population is correlated with enhanced HER activity [17–21]. In this context, to modify the hydrogen-bonding network, several studies have explored the use of hetero-solvents in various aqueous electrochemical systems [21–26]. In these systems, hetero-solvents in bulk aqueous electrolyte can form new hydrogen bonding with H_2_O, reduce the free-water population, and consequently suppress HER. For example, incorporating dimethylformamide (DMF) as a hetero-solvent significantly reduced HER during CO_2_RR to CO on gold catalyst in an aqueous H-cell [23]. *In situ* surface-enhanced infrared absorption spectroscopy (SEIRAS) in attenuated total reflection (ATR) mode revealed a substantial decrease in free-water formation in the presence of DMF. Likewise, the incorporation of hetero-solvents has been reported to inhibit free-water formation and thereby mitigate competing HER in alkenol electrosynthesis [21] and aqueous battery systems [25, 26]. However, use of hetero-solvents in bulk electrolyte is typically constrained at high current densities by increased cell voltages arising from their high viscosity and low ionic conductivity [22, 27, 28].

Beyond influencing HER, hydrogen-bonding network of interfacial water also affects the hydrogenation of CO_2_RR intermediates, thereby altering product selectivity. The hydrogenation of adsorbed intermediates can proceed *via* two distinct mechanisms: the Langmuir–Hinshelwood (LH) mechanism, which involves a surface reaction between two adsorbed species of *H and CO_2_RR intermediate, or the Eley–Rideal (ER) mechanism, in which only the CO_2_RR intermediate is adsorbed while H is directly supplied through water dissociation in the solvent phase (solvent hydrogenation) [29, 30]. Recent isotopic labeling and theoretical studies showed that ethanol formation prefers LH hydrogenation, whereas ethylene formation is favored by solvent-mediated ER hydrogenation [29–32]. These insights suggest that modulating hydrogen-bonding network of interfacial water can steer the selectivity between ethanol and ethylene.

Ethanol is a particularly attractive product due to its ease of storage and transport, as well as its versatile applications as solvent, fuel blend, and raw material for various chemicals [33, 34]. Consequently, intensive research efforts have been devoted to enhancing ethanol selectivity during CO_2_RR on Cu, primarily by tuning the binding energies of C_2+_ intermediates through catalyst design, such as doping and alloying [30, 35–37]. However, surface reconstruction easily occurs during CO_2_RR, making it challenging to maintain initial performance [38–40]. Another issue in ethanol production during CO_2_RR lies in the efficient recovery of ethanol. Since produced ethanol is in liquid electrolyte, additional separation processes such as distillation are required. Many previous studies achieving considerable ethanol yields have employed alkaline electrolytes. However, ethanol in alkaline conditions can form acetaldehyde due to its lower chemical stability compared to neutral conditions [41, 42]. Additionally, the aldol condensation reaction can occur during the high-temperature distillation process under alkaline condition [43, 44]. Moreover, the higher viscosity of alkaline electrolytes reduces distillation efficiency [45–47].

Here, we propose a strategy to modulate the hydrogen-bonding network of interfacial water under neutral condition using a hetero-solvent. As the hetero-solvent, we selected diglyme (DiG), bis(2-methoxyethyl) ether, based on three key criteria essential for an effective hetero-solvent under CO_2_RR conditions: (i) high CO_2_ solubility to alleviate mass transport limitations within catalytic microenvironment, (ii) hydrogen-bonding accepting yet aprotic character to restructure the interfacial water network without introducing additional proton sources that would promote HER, and (iii) electrochemical stability under cathodic conditions relevant to CO_2_RR, as shown in Table S1. However, DiG has higher viscosity and cost than water; thus, its incorporation in bulk electrolyte can increase both cell voltage and the cost. In this regard, we aim to selectively tune the water network within the catalytic microenvironment, where its influences on HER and CO_2_RR are most pronounced, *via* confining the DiG hetero-solvent using ionomer coating, which can modulate ion and mass transport including small molecules within the microenvironment.

## Experimental Section

### Materials Characterization

The chemical states of the electrode surface were examined by XPS (ESCALAB 250 Xi, Thermo Fisher Scientific). The XPS depth profiles were collected by 4 rounds of Ar etching, with an interval time of 200 s. The XPS spectra were calibrated using the C 1*s* reference to 284.6 eV. The crystal structures of electrodes were analyzed by the powder XRD using Rigaku Miniflex 600 with Cu Kα radiation (λ = 1.54056 Å) at 40 kV accelerating voltage with a scan rate of 2° min^−1^. NMR (AVANCE III 400, Bruker) analysis was carried out to investigate the dissolution of DiG during the CO_2_RR.

### Electrode Preparation

The Cu GDE was fabricated on a carbon paper (Sigracet 39BB, FUELCELL Store) and PTFE (Sterlitech) using a DC magnetron sputtering system (DDHT-SS3R4). To prepare the Naf/Cu electrode, Nafion ionomer (Chemours, 5 wt%) mixed with IPA was spray-coated onto 300 nm sputtered Cu using an airbrush gun with N_2_ flow, resulting in a Nafion loading mass of 57.5 µg cm^−2^. To prepare the Naf/DiG/Cu, DiG mixed with IPA was loaded onto 300 nm sputtered Cu, resulting in a DiG loading mass of 57.5 µg cm^−2^, followed by Nafion coating using the same procedure as for Naf/Cu. After sample preparation, the electrodes were dried at room temperature for 12 h under vacuum conditions.

### Electrochemical Measurements

The CO_2_RR measurements were conducted using catholyte-free MEA electrolyzer (Dioxide Materials; 5 cm^2^ electrode area) based on a 1 M KHCO_3_ (99.7%, Sigma-Aldrich) anolyte. IrO_x_/Ti (2GDL6N-025 BS20IR; Bekaert; 2 mg cm^−2^ loading) was used as counterelectrode. Sustainion X37-50 (Dioxide Materials) was used as anion exchange membrane (AEM) after activation in 1 M KOH for 24 h followed by washing in DI water. The MEA electrolyzer was compressed by a torque wrench with a torque of 3 Nm. The electrolyte was circulated on the anode side using a peristaltic pump with 20 sccm, whereas humidified CO_2_ gas (99.999%) was supplied to the cathode side using mass flow controller (ALICAT Scientific) with a constant flow rate of 50 sccm. Full cell voltage was applied to the electrolyzer by a potentiostat with current booster (PGSTAT204, Autolab). Gas products collected by gas-tight syringe and liquid products collected from the anolyte and cold trap were measured by gas chromatography (Clarus 690, PerkinElmer) and NMR with dimethyl sulfoxide (Sigma-Aldrich) as an internal standard, respectively. The Faradaic efficiency of the resultant value was derived from the following equation:

$${\mathrm{FE}}\left( \% \right) = \frac{z \cdot n \cdot F}{Q}$$

where z and n are the number of electrons exchanged and moles of products; F is the Faradaic constant; Q is input charge.

### *In Situ* SEIRAS Measurements

The Cu nanofilm was prepared on the total reflecting plane of a hemispherical Si ATR prism (radius 20 mm) using the slightly modified electroless deposition method reported by Osawa et al. [48]. Prior to electroless Cu deposition, the prism was mechanically polished with a diamond suspension (1 µm, Allied High Tech Products Inc.) and then immersed in 40 wt% NH_4_F aqueous solution (98%, Sigma-Aldrich) for 1 min to render the surface hydrophilic. To introduce the Cu seeds on the surface, the prism was subsequently immersed for 10 s in a Cu seeding solution prepared by dissolving 3.15 mM CuSO_4_·5H_2_O (98%, Sigma-Aldrich) in 0.625 M HF (48%, Sigma-Aldrich). After rinsing with DI water, the electroless Cu deposition was carried out by immersing the prism twice (10 min each) in Cu deposition solution. The Cu deposition solution was prepared by mixing 0.02 M CuSO_4_·5H_2_O (98%, Sigma-Aldrich), 0.09 M C_4_H_4_O_6_KNa·4H_2_O (99%, Sigma-Aldrich), 10 mL L^−1^ HCHO (37 wt%, Sigma-Aldrich), and NaOH (99.99%, Sigma-Aldrich), adjusted to pH 12.7. All deposition steps were conducted at room temperature. The prepared Cu-deposited Si prisms were dried at room temperature for 12 h under vacuum condition.

To introduce hetero-solvent microenvironment, DiG and Nafion were sequentially spray coated onto vacuum-dried Cu-deposited Si prism. DiG (≥ 99%, Sigma-Aldrich) with 2-propanol (IPA) was sprayed using an air brush gun, yielding a DiG loading of 57.5 µg cm^−2^. Subsequently, Nafion ionomer (5 wt%, DuPont) with IPA was similarly sprayed to achieve a Nafion loading of 57.5 µg cm^−2^. The Naf/DiG/Cu prism was used after coating immediately.

*In situ* SEIRAS spectra were collected using a Nicolet iS50 (Thermo Fisher Scientific) equipped with a liquid nitrogen-cooled HgCdTe (MCT) detector. The optical path was purged with N_2_ gas (99.999%, Donghae Gas Ind.) to eliminate atmospheric H_2_O and CO_2_ interferences. Spectra were measured using a specular reflection unit (VeeMax III, PIKE Technologies) paired with a Si ATR prism and a light polarizer with the incident angle set to 67°. Spectra were recorded at 8 cm^−1^ resolution by averaging 32 scans per spectrum. The geometric area of the Cu working electrode was 0.5 cm^2^. The O–H stretching mode was monitored during LSV from − 0.3 to − 1.0 V_RHE_ at a scan rate of 1 mV s^−1^ in CO_2_-saturated 1 M KHCO_3_ (99.7%, Sigma-Aldrich). Reference spectrum was gathered at 0.3 V_RHE_ in CO_2_-saturated 1 M KHCO_3_.

### Computational Details

All ab initio molecular dynamics (AIMD) simulations were carried out using the Vienna Ab initio Simulation Package (VASP) [49], projected augmented wave (PAW) [50] pseudopotential method, and Perdew–Burke–Ernzerhof (PBE) exchange–correlation functional [51]. A plane-wave cutoff energy of 400 eV was applied. The convergence criteria for energy and force were set to 10–4 eV and 0.05 eV Å^−1^, respectively. Furthermore, van der Waals interactions were included using Grimme's DFT-D3 dispersion correction scheme [52, 53]. All AIMD simulations were performed with a 1 fs time step, and the Brillouin zone was sampled at the gamma point. All runs were conducted under the canonical ensemble (NVT) at a target temperature of 300 K, controlled by a Nosé–Hoover thermostat with symmetry disabled.

To investigate the structure of interfacial water molecules, the vibrational density of states (VDOS) was analyzed. Water molecules were first placed on the Cu (111) surface and equilibrated for 10 ps. Snapshots from the subsequent 5-ps simulation trajectories were then extracted for the VDOS analysis, which was post-processed using VASPKIT [54].

To evaluate the free energy barriers of LH and ER mechanisms on the DiG/Cu surface, H* and HCCOH* adsorbates were initially positioned on the surface and equilibrated for 10 ps. The free energy barriers for both the LH and ER mechanisms were subsequently evaluated using the collective variable (CV) method. For the ER mechanism, the CV was defined as the difference between distances ${\mathrm{r}}_{1} = {\text{ O}}_{1} { - }{\mathrm{H}}$ and ${\mathrm{r}}_{2} = {\text{ O}}_{1} { - }{\mathrm{C}}$ (${\text{CV = r}}_{1} - {\mathrm{r}}_{2}$), where ${\mathrm{O}}_{1}$ and C refer to atoms of HCCOH* and H refers to the H atom of a neighboring water molecule, respectively. For the LH mechanism, the CV was defined as the distance between the C atom and H* (${\text{CV = r}}_{{1}} {\text{ = C}} - {\mathrm{H}}$).

## Results and Discussion

### Design of Hetero-Solvent Microenvironment

**Fig. 1:**
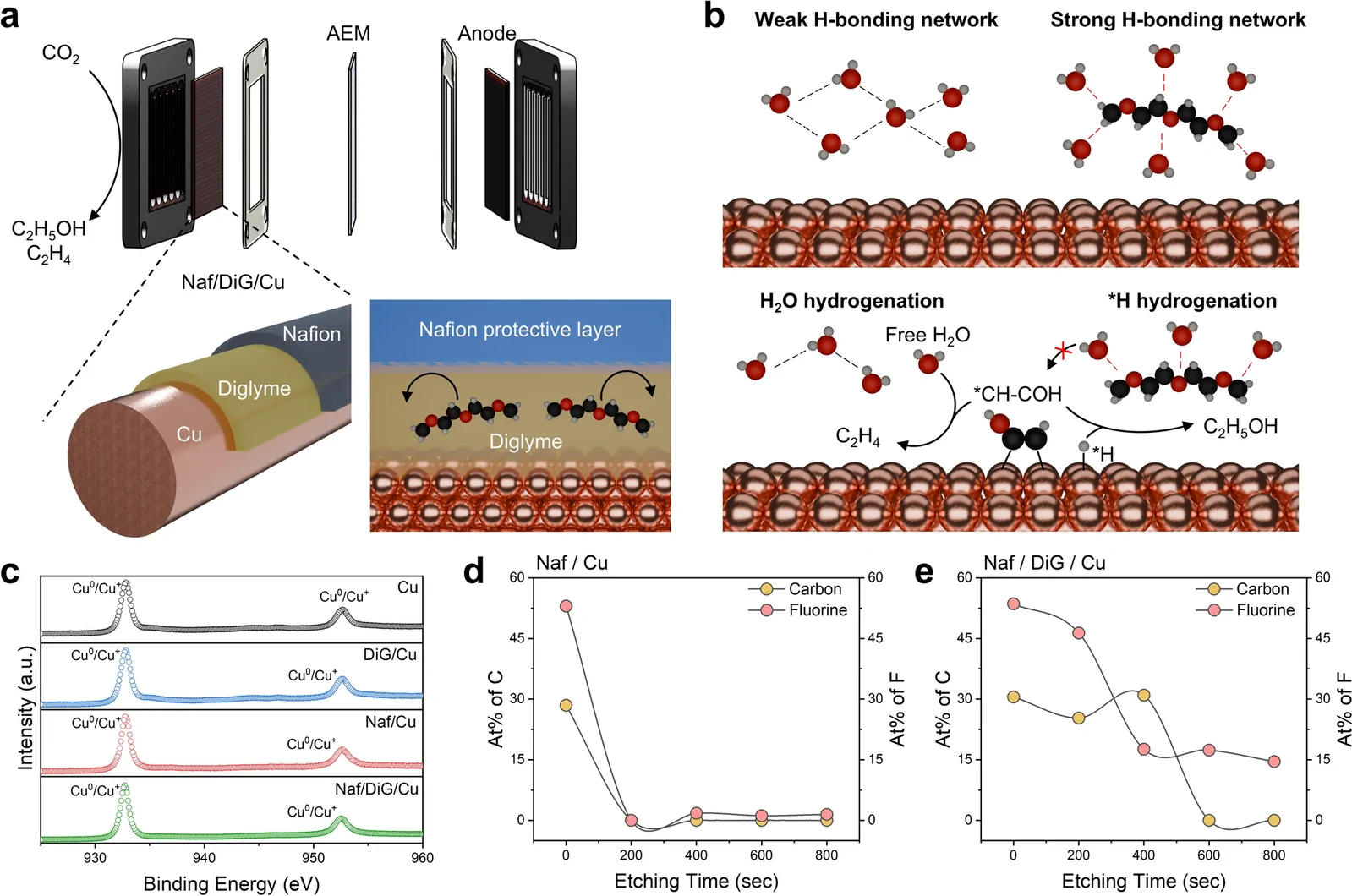
Characterization of hetero-solvent-incorporated Cu electrode. **a** Design of MEA CO_2_ electrolyzer incorporating Naf/DiG/Cu electrode. **b** Hydrogen-bonding network regulation *via* hetero-solvent to suppress HER and promote CO_2_-to-C_2_H_5_OH conversion. **c** XPS spectra of Cu 2*p* for Cu, DiG/Cu, Naf/Cu, and Naf/DiG/Cu. XPS depth profile of C 1*s* and F 1*s* for **d** Naf/Cu and **e** Naf/DiG/Cu

First, DiG hetero-solvent was incorporated into microenvironment near Cu catalyst in a membrane electrode assembly (MEA) (Fig. 1a). Specifically, a Nafion/diglyme/Cu (Naf/DiG/Cu) electrode was designed to confine the DiG within the microenvironment. In this cathode design, Nafion serves as a protective layer to prevent the leakage of DiG during CO_2_RR. Additionally, Nafion ionomers can enhance CO_2_-to-C_2+_ electro-conversion by increasing local pH at the catalyst–electrolyte interface. Owing to the presence of a negatively charged group (SO_3_^−^), Nafion effectively confines OH^−^ anions generated during CO_2_RR, thereby promoting a locally alkaline environment that favors C_2+_ production [55]. Based on this rationale, Nafion was selected in this study rather than AEIs, such as Sustainion, which does not provide OH^−^ confinement or local pH modulation. The confined DiG can modulate the hydrogen-bonding network of water in microenvironment (Fig. 1b). As a result, Naf/DiG/Cu electrode would minimize the population of free-water, thereby suppressing HER. In addition, the restricted formation of free-water may alter the hydrogenation pathways of CO_2_RR intermediate, such as *CH–COH for ethanol or ethylene formation [30].

To investigate characteristics of the prepared Naf/DiG/Cu electrodes, X-ray photoelectron spectroscopy (XPS) was conducted (Fig. 1c–e). There were no noticeable differences in XPS spectra of Cu 2*p* for Cu, Naf/Cu, and Naf/DiG/Cu samples, indicating that the incorporation of Nafion and DiG does not alter the electronic structure of the Cu surface (Fig. 1c). In addition, X-ray diffraction (XRD) patterns and scanning electron microscopy (SEM) images also confirmed no considerable change in Cu crystalline and morphological structure for all three samples (Figs. S1 and S2).

Additionally, XPS depth profiles of C 1*s* and F 1*s* were obtained to verify the existence of DiG between Cu electrode and Nafion layer in prepared Naf/DiG/Cu (Fig. 1d, e). In Naf/Cu (Nafion-coated Cu), the atomic percentages of C and F decreased rapidly upon Ar sputtering, reflecting the progressive removal of the thin Nafion overlayer by ion etching and the subsequent exposure of the underlying Cu surface (Fig. 1d). In contrast, the Cu 2*p* spectra appeared upon Ar etching, indicating that the underlying Cu layer was exposed (Fig. S3b). These XPS depth profile results for Naf/Cu demonstrate that Nafion remains as a thin layer on the Cu surface after electrode fabrication. By contrast, the Naf/DiG/Cu electrode exhibited a more gradual decrease in atomic percentages of C and F upon Ar etching, along with a bump in the C profile and a saddle point in the F profile, confirming a more swollen Nafion layer and the presence of DiG between the Nafion and Cu layers, respectively (Figs. 1e and S4). In addition, electrochemical impedance spectroscopy (EIS) analysis was conducted to evaluate the effect of DiG on charge transfer properties. The Nyquist plots show comparable solution resistance (R_s_) values of 1.6 and 1.7 Ω for Cu and Naf/DiG/Cu, respectively (Fig. S5). The EIS results suggest that the Naf/DiG layer has a negligible impact on intrinsic reaction kinetics and cell resistance.

Furthermore, nuclear magnetic resonance (NMR) analysis was performed to examine whether any leakage of DiG occurred from the designed microenvironment during CO_2_RR. As a control group, NMR spectrum obtained after immersing the DiG/Cu electrode in deionized (DI) water for 30 min reveals the peak corresponding to DiG, indicating that DiG easily dissolves in water in the absence of Nafion protective layer (Fig. S6). In contrast, the NMR spectra of anolyte and cold trap after CO_2_RR at 2.7 and 3.6 V using the Naf/DiG/Cu electrode showed no peaks corresponding to DiG, demonstrating that the Nafion layer effectively prevents the leakage of DiG into water (Fig. S7). In addition, when the used MEA after CO_2_RR was deliberately torn apart and immersed in water, the NMR peak corresponding to DiG was observed, indicating preservation of DiG within the microenvironment after CO_2_RR (Fig. S8). To exclude the possibility of DiG decomposition during CO_2_RR, we performed online differential electrochemical mass spectrometry (DEMS) analysis. The m/z = 31 signal was monitored as a characteristic fragment that could arise from C–O bond cleavage of DiG. Notably, this signal also corresponds to the primary mass fragment of ethanol (Fig. S9). To verify these possibilities, we monitored the m/z = 31 signal at 3.6 V under an Ar flow. As shown in Fig. S10, no detectable signal was observed under cathodic bias over 30 min. This online DEMS result provides additional evidence for the stability of the DiG layer in the Naf/DiG/Cu electrode and confirms that ethanol is not generated from DiG decomposition. These results clearly show that the designed Naf/DiG/Cu electrode was stable and effectively confined the hetero-solvent within the microenvironment.

### Hetero-Solvent Effects on CO_2_RR

**Fig. 2:**
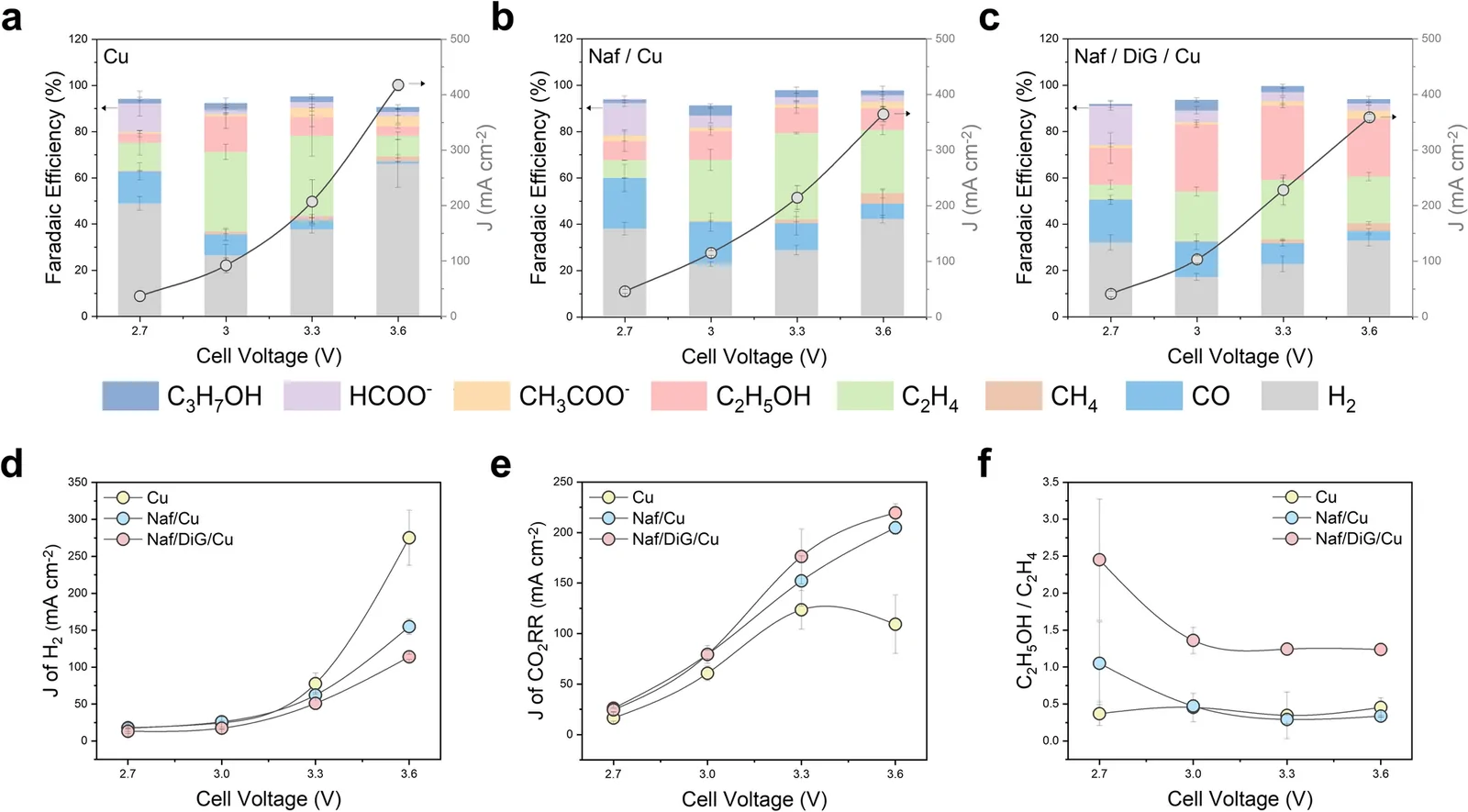
Effects of DiG on CO_2_RR activity and selectivity. CO_2_RR performance of **a** Cu, **b** Naf/Cu, and **c** Naf/DiG/Cu. Partial current densities for **d** H_2_ and **e** CO_2_RR of Cu, Naf/Cu, and Naf/DiG/Cu. **f** Selectivity of C_2_H_5_OH compared to C_2_H_4_ of Cu, Naf/Cu, and Naf/DiG/Cu

To evaluate the effect of hetero-solvent, CO_2_RR was performed in the MEA electrolyzer with 1 M KHCO_3_ anolyte at various applied cell voltages (Fig. 2a–c). At low cell voltage, mass transport limitations are minimal leading to the similar CO_2_RR performance. As the potential increases, the rapid consumption of CO_2_ induces concentration gradients and diffusion limitations, thereby amplifying the influence of mass transport and leading to a pronounced difference in catalytic performance [48, 56]. With increasing cell voltage, the bare Cu electrode showed an increased Faradaic efficiency (FE) for HER, indicating increased selectivity toward HER over CO_2_RR (Fig. 2a). Conversely, the Naf/Cu electrode showed a suppressed HER, which can be attributed to the enhanced hydrophobicity in the presence of the Nafion layer (Fig. 2b) [57]. As shown in Fig. S11, the contact angle of the catalytic surface increases upon incorporation of a Nafion layer, indicating enhanced surface hydrophobicity. This increased hydrophobicity would improve CO_2_RR performance by suppressing the competing HER. Notably, the Naf/DiG/Cu electrode exhibited the lowest FE for HER across the entire cell voltage and consequently the highest FE for CO_2_RR (Fig. 2c).

The partial current densities representing activity toward HER and CO_2_RR are shown in Fig. 2d, e. The bare Cu exhibited the highest H_2_ partial current density of 275.1 mA cm^−2^ at a cell voltage of 3.6 V, which is 2.4-fold higher than that of Naf/DiG/Cu electrode (Fig. 2d). The HER partial current density was lowest in the presence of DiG in microenvironment (Naf/DiG/Cu), indicating the most mitigated HER activity. Additionally, the Naf/DiG/Cu electrode achieved CO_2_RR partial current density of 219.6 mA cm^−2^, which is 2.0-fold higher than that of bare Cu (Fig. 2e). Thus, the Naf/DiG/Cu showed the highest CO_2_RR activity among the tested samples, which is likely associated with the enhanced CO_2_ solubility in DiG (160 mM), compared with that in water (34 mM) and Nafion (36.1 mM) [4].

Regarding selectivity among CO_2_RR products, it is particularly notable that ethanol and ethylene selectivities were substantially altered in the presence of DiG in the microenvironment (Fig. 2f). This observation is particularly intriguing because the Cu electrode typically favors production of ethylene over ethanol during CO_2_RR. Although Naf/Cu showed noticeable HER suppression due to increased hydrophobicity, its FE ratio of ethanol to ethylene closely resembled that of bare Cu. On the other hand, Naf/DiG/Cu electrode showed substantially higher ethanol-to-ethylene FE ratio than the others across all tested cell voltages. Based on these observed effects in the presence of DiG, suppressing HER and enhancing ethanol selectivity, we hypothesize that the incorporation of DiG reinforces the hydrogen-bonding network of interfacial water, thereby suppressing HER and altering the hydrogenation pathway of the C_2_ intermediate. This hypothesis aligns well with recent reports suggesting that the hydrogenation pathway, whether mediated by free H_2_O or *H, is a key determinant of ethylene versus ethanol production during CO_2_RR on Cu surface [30]. Accordingly, the remaining questions concern how DiG modulates the hydrogen-bonding network of interfacial water and how this modulation governs the hydrogenation of C_2+_ intermediates.

### Hydrogen-Bonding Network of Interfacial Water in the Presence of Hetero-Solvent

**Fig. 3:**
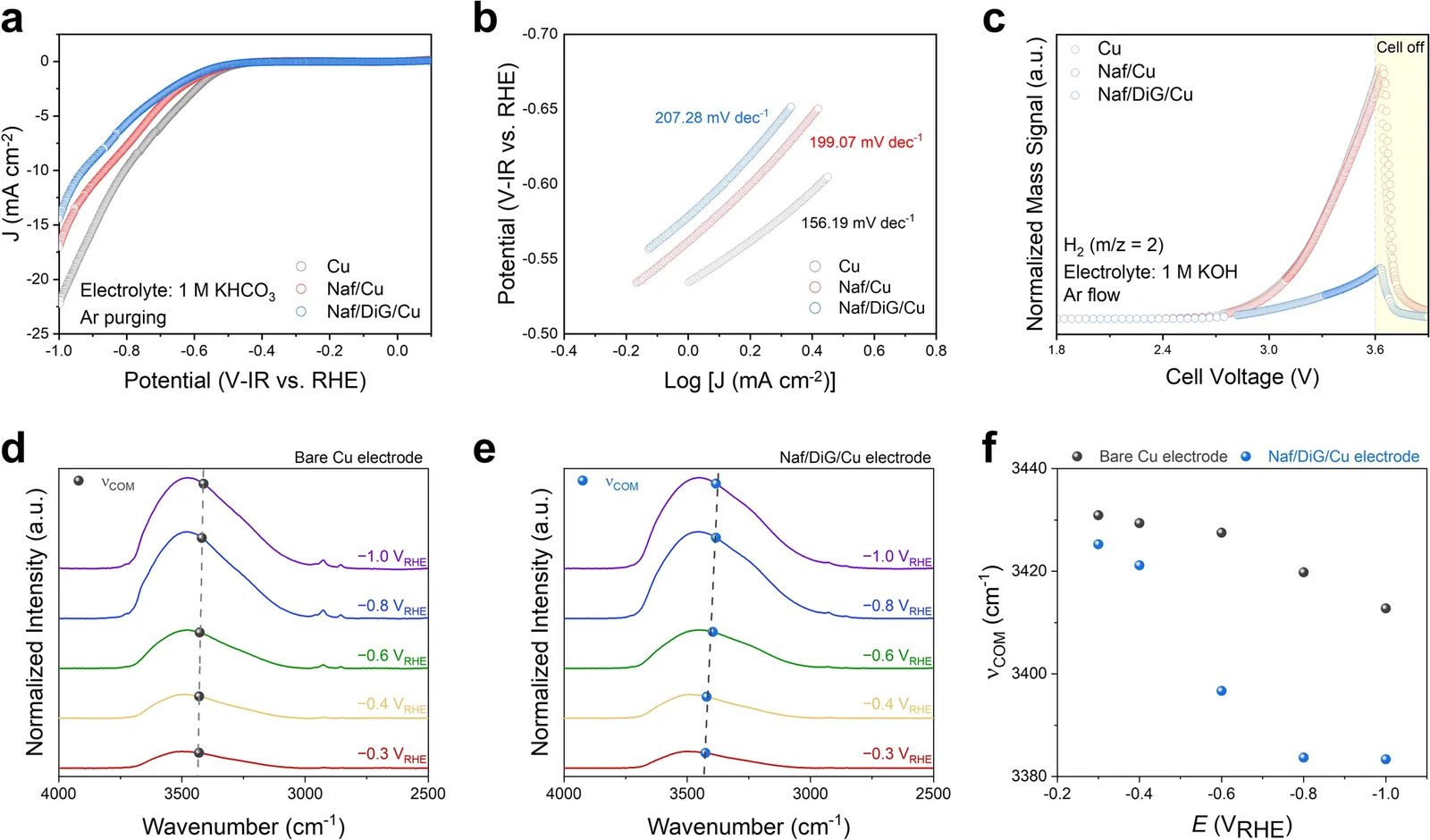
DiG effects on hydrogen-bonding network of interfacial water. **a** LSV curves with Ar purging of Cu, Naf/Cu, and Naf/DiG/Cu. **b** TSs for HER of Cu, Naf/Cu, and Naf/DiG/Cu. **c** Online DEMS measurements for H_2_ of Cu, Naf/Cu, and Naf/DiG/Cu. *In situ* SEIRAS spectra on **d** Cu and **e** Naf/DiG/Cu electrodes. The ν_COM_ values are indicated with dots. **f** Correlations of ν_COM_ values for Cu and Naf/DiG/Cu with applied potentials

To decouple the effect of DiG on HER from CO_2_RR processes, HER was independently investigated by linear sweep voltammetry (LSV) in CO_2_-free, Ar-purged 1 M KHCO_3_ (Fig. 3a). Among all samples, the bare Cu electrode exhibited the highest HER activity, with an overpotential (η) of − 0.67 V at 5 mA cm^−2^ and a Tafel slope (TS) of 156.19 mV dec^−1^ (Fig. 3b). On the other hand, the Naf/DiG/Cu electrode exhibited an η of − 0.78 V and a TS of 207.28 mV dec^−1^, indicating that the introduction of Naf/DiG significantly suppressed HER under neutral conditions. This suppression was further corroborated by online DEMS (Fig. 3c) in Ar-purged 1 M KOH, which revealed diminished H_2_ signals (m/z = 2) during polarization on Naf/DiG/Cu compared with other electrodes. The impact of modulating the hydrogen-bonding network is expected to be more pronounced than that under Ar-purged 1 M KHCO_3_ conditions, because HER in Ar-purged 1 M KOH predominantly proceeds *via* dissociation of free H_2_O, whereas buffer-mediated proton transfer may contribute under Ar-purged 1 M KHCO_3_ [58]. These DEMS results support that the modulated hydrogen-bonding network in the presence of DiG efficiently limits the dissociation of free H_2_O, thereby suppressing HER.

Given the increase in TS value under neutral condition and DEMS result under alkaline condition, it can be inferred that the introduction of the Naf/DiG layer considerably hinders the rate-determining Volmer step during HER under neutral and alkaline condition, which involves a water dissociation step [59]. To elucidate the underlying causality between the hydrogen-bonding network of interfacial water and the observed trends in HER and CO_2_RR, we monitored the O–H stretching vibration (ν = 3000–3700 cm^−1^) of interfacial water molecules using *in situ* SEIRAS on Cu-deposited Si ATR prism with and without the Naf/DiG layer. The spectra were collected in CO_2_-saturated 1 M KHCO_3_ in a potential range from − 0.3 to − 1.0 V_RHE_ (Fig. 3d, e). To quantitatively assess the hydrogen-bonding strength without the ambiguity of conventional fitting (Fig. S12), the center of mass (ν_COM_) of the broad O–H stretching band was estimated at each applied potential. The results showed that the ν_COM_ for Naf/DiG/Cu shifted progressively to lower wavenumbers with increasing negative potentials (Fig. 3f), whereas the shift on bare Cu was much less pronounced. This indicates that the Naf/DiG layer promotes a more ordered hydrogen-bonding network within the interfacial water layer. Considering previous findings that a more disordered hydrogen-bonding network facilitates proton transfer from water [60], the suppressed HER activity on Naf/DiG/Cu can thus be attributed to the formation of an ordered interfacial water network.

### Modulation of Ethanol Production in the Presence of Hetero-Solvent

**Fig. 4:**
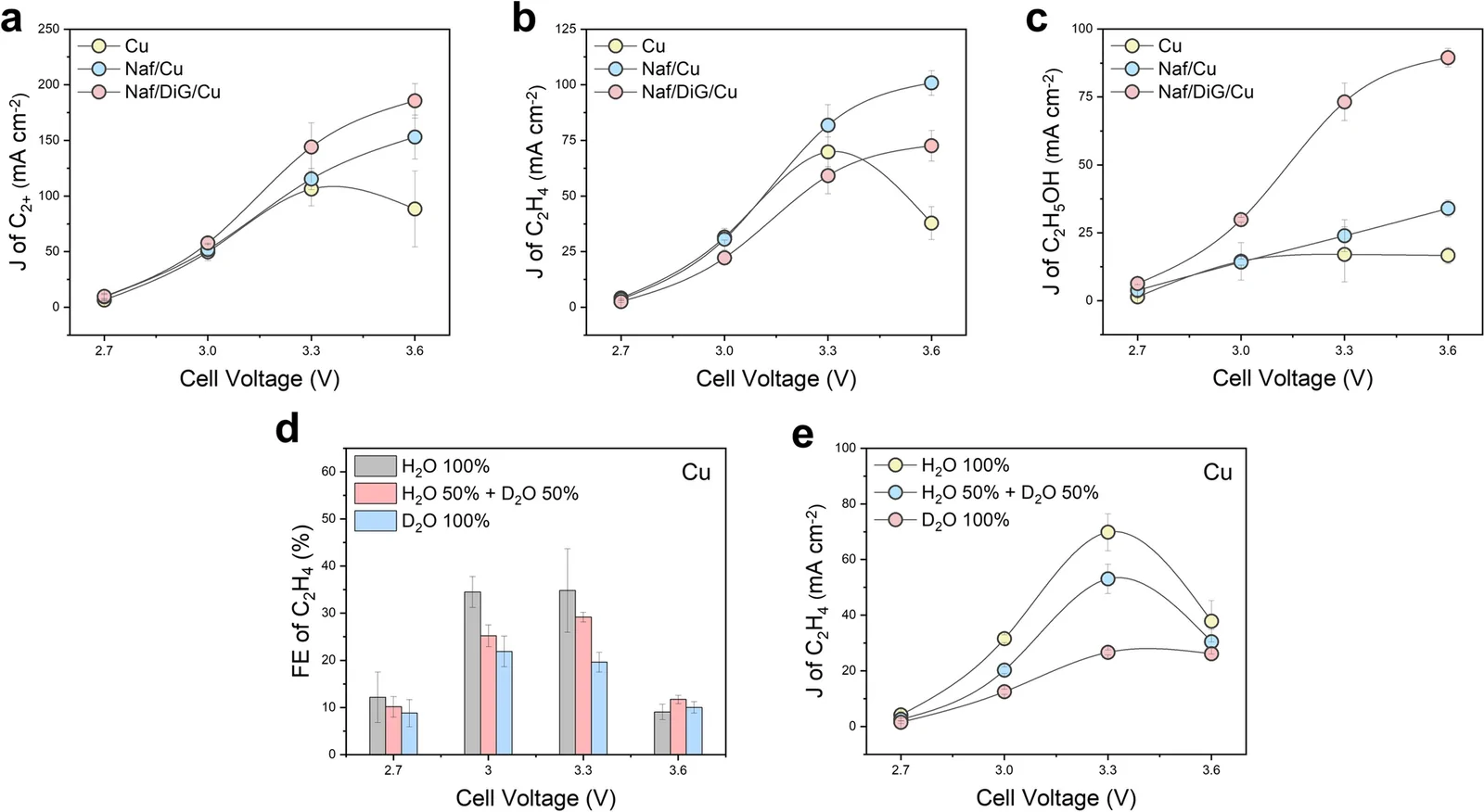
Correlation between C_2_H_5_OH/C_2_H_4_ selectivity and hydrogen-bonding network. Partial current densities for overall **a** C_2+_, **b** C_2_H_4_, and **c** C_2_H_5_OH of Cu, Naf/Cu, and Naf/DiG/Cu. **d** FE and **e** partial current densities of C_2_H_4_ depending on the H_2_O/D_2_O ratio

The Naf/DiG/Cu electrode, in which the hydrogen-bonding network of interfacial water is modulated, not only promotes the CO_2_ to C_2+_ conversion but also governs the selectivity between ethylene and ethanol (Fig. 4a–c). For the bare Cu electrode, the partial current density of C_2+_ products noticeably decreased at a cell voltage of 3.6 V, likely due to the rapid increase in HER at 3.6 V (Fig. 4a). Conversely, the partial current densities for C_2+_ products on Naf/Cu and Naf/DiG/Cu electrodes increased continuously with increasing cell voltage. These results can be attributed to the enhanced hydrophobicity in the presence of the Nafion layer and improved CO_2_ solubility in the presence of DiG. The partial current densities of the major C_2_ products, ethylene and ethanol, are shown in Fig. 4b, c. For the bare Cu electrode, the C_2_H_4_ partial current density increased with increasing cell voltage, but decreased at 3.6 V (Fig. 4b). The Naf/Cu electrode showed an increased C_2_H_4_ partial current density with increasing cell voltage, owing to HER suppression compared to bare Cu. On the other hand, the Naf/DiG/Cu electrode presented the lowest C_2_H_4_ partial current density, which is attributed to a selectivity shift from C_2_H_4_ to C_2_H_5_OH. Notably, the C_2_H_5_OH partial current density of Naf/DiG/Cu reached 89.5 mA cm^−2^ at 3.6 V, which is 2.6- and 5.3-fold higher than those of Naf/Cu and bare Cu, respectively (Fig. 4c). Collectively, these findings demonstrate that while bare Cu and Naf/Cu intrinsically favor C_2_H_4_ formation, the Naf/DiG/Cu fundamentally redirects C_2_ product selectivity toward C_2_H_5_OH.

Recent computational studies have revealed that hydrogenation of the C atom primarily proceeds *via* surface hydrogenation, whereas hydrogenation of the O atom occurs mainly through a solvent-mediated pathway. These mechanistic differences govern the differentiation of C_2_ intermediate into C_2_H_5_OH and C_2_H_4_, respectively [29, 30]. Furthermore, a recent isotope labeling study has demonstrated that increased water dissociation promotes C_2_H_4_ formation by facilitating solvent-mediated hydrogenation [61]. In this regard, to investigate the relationship between hydrogen-bonding network of interfacial water and C_2_ selectivity (C_2_H_4_ vs. C_2_H_5_OH), we performed the isotope labeling analysis, as shown in Fig. 4d, e. Consistent with the previous literature, the selectivity and activity of C_2_H_4_ formation were significantly influenced by the H_2_O/D_2_O ratio in our experiment. Specifically, HER activity decreased with increasing D_2_O concentration due to the suppression of water dissociation, which in turn inhibits the Volmer step (Fig. S13). Alongside HER, increasing D_2_O content also led to a decrease in both the selectivity and activity of C_2_H_4_ formation (Fig. 4d, e). Results from isotope labeling experiments indicate that the rate-determining steps of HER and C_2_H_4_ formation associate with a proton transfer from water, which is thus expected to be strongly affected by the hydrogen-bonding network of interfacial water. Consequently, these results support our hypothesis that the Naf/DiG/Cu electrode inhibits water dissociation, thereby hindering C_2_H_4_ formation *via* solvent-mediated hydrogenation, which in turn facilitates C_2_H_5_OH formation.

### Theoretical Insights into the Hetero-Solvent Effect on CO_2_RR Hydrogenation Pathway

Ab initio molecular dynamics (AIMD) simulations were performed to provide theoretical insights into how DiG modulates the interfacial water network and how this modulation selectively affects the LH and ER mechanisms.

**Fig. 5:**
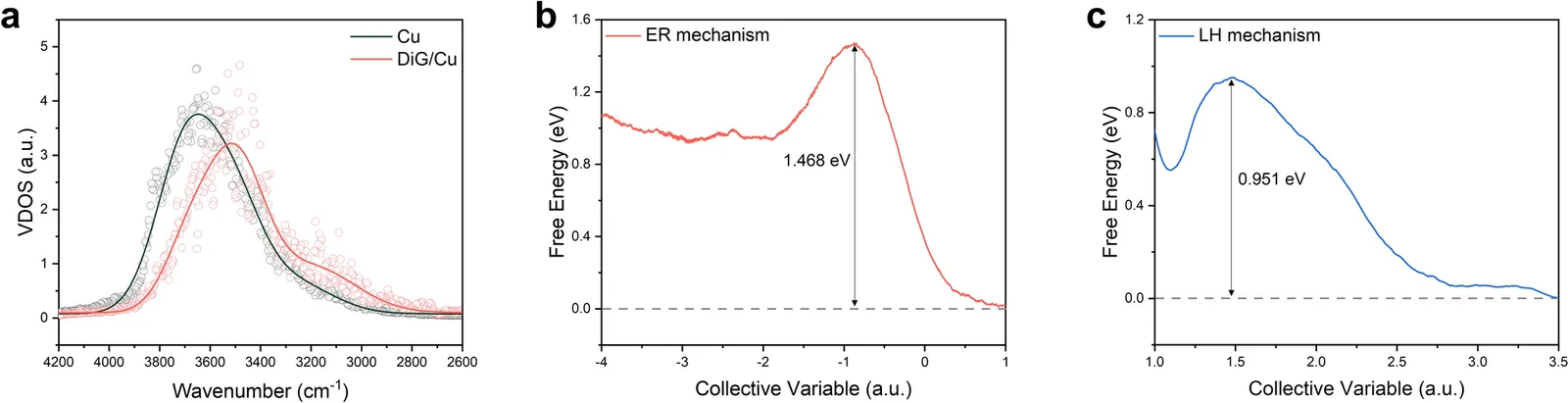
**a** Vibrational density of state (VDOS) profiles of interfacial water molecules on Cu and DiG/Cu surfaces, computed from AIMD simulations. Gaussian fitting was applied to smooth the resulting curves. Free energy profiles obtained from constrained slow-growth AIMD simulations for the **b** ER and **c** LH mechanisms

First, vibrational density of states (VDOS) profiles were calculated to analyze the interfacial water structure on both bare Cu and DiG/Cu surfaces. As shown in Fig. 5a, the computed O–H stretching band of interfacial water molecules on DiG/Cu shifts to lower wavenumbers compared to that on bare Cu. This shift indicates a stronger hydrogen-bonding network in the presence of DiG. This result is in good agreement with *in situ* SEIRAS results (Fig. 3d–f), in which a similar shift of ν_COM_ was observed in the presence of DiG.

Building on this, slow-growth AIMD simulations were conducted to evaluate how DiG-induced modulation of the interfacial water network kinetically differentiates the ER and LH mechanisms. Since HCCOH* has been widely recognized as the shared key intermediate from which the ethylene and ethanol pathways branch [62, 63], we examined the free energy barriers for its hydrogenation *via* each mechanism (Fig. S14). The results show that the free energy barrier of the ER mechanism (1.468 eV) significantly exceeds that of the LH mechanism (0.951 eV) (Fig. 5b, c) in the presence of DiG. These results indicate that solvent hydrogenation, which preferentially leads to C_2_H_4_ formation, is kinetically suppressed in the presence of DiG, while the LH pathway, which preferentially leads to C_2_H_5_OH formation, becomes the dominant route. This computational finding is fully consistent with the experimentally observed shift in C_2_ selectivity from C_2_H_4_ to C_2_H_5_OH on Naf/DiG/Cu, as well as with the D_2_O isotope labeling experiments in Fig. 4d, e.

### Scalability of Hetero-Solvent Microenvironment

**Fig. 6:**
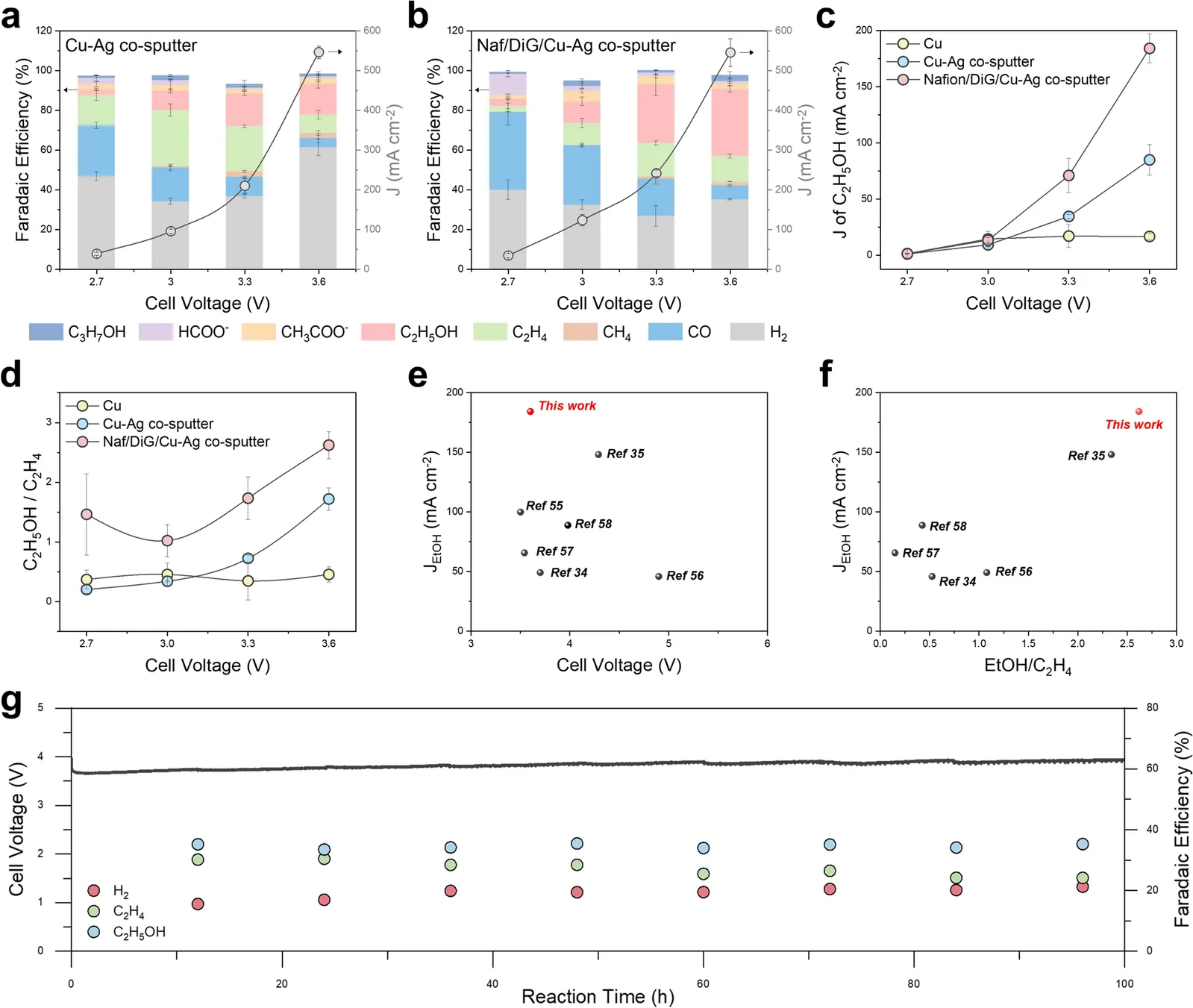
CO_2_RR performance of hetero-solvent-incorporated Cu-Ag bimetallic electrode. CO_2_RR performance of **a** Cu-Ag and **b** Naf/DiG/Cu-Ag electrodes. **c** Partial current densities for C_2_H_5_OH of Cu-Ag and Naf/DiG/Cu-Ag. **d** Selectivity of C_2_H_5_OH compared to C_2_H_4_ of Cu-Ag and Naf/DiG/Cu-Ag. **e–f** Comparison with state-of-the-art literature on ethanol production by MEA CO_2_RR in neutral media [33, 34, 66–69]. **g** Long-term stability measurements of Naf/DiG/Cu under 150 mA cm^–2^ for 100 h

The proposed strategy is based on engineering of the microenvironment which is independent on intrinsic properties of the catalytic material. Thus, this strategy can be readily scaled to other systems with various catalytic materials, enabling synergistic effects. In this regard, we applied the hetero-solvent microenvironment to a Cu-Ag bimetallic catalyst (Fig. 6a, b). Previous studies have reported that the Cu-Ag bimetallic catalysts can improve C_2_H_5_OH production by promoting the asymmetric binding of CO-CH_x_ or CO-CHO intermediates [64, 65]. Consistent with these reports, the Cu-Ag co-sputtered electrode exhibited a higher partial current density for C_2_H_5_OH compared to bare Cu (Fig. 6c). Notably, incorporating the hetero-solvent microenvironment into the Cu-Ag electrode (Naf/DiG/Cu-Ag) further enhanced C_2_H_5_OH formation while simultaneously suppressing HER, demonstrating that DiG-induced interfacial water control is effective for Cu-Ag bimetallic system as well (Figs. 6c and S15, S16). Specifically, the C_2_H_5_OH partial current density of Naf/DiG/Cu-Ag reached 184.2 mA cm^−2^ at 3.6 V, corresponding to 2.2-fold and 11.1-fold enhancements relative to bare Cu-Ag and bare Cu electrodes, respectively (Fig. 6c). Additionally, the selectivity of C_2_H_5_OH relative to C_2_H_4_ was markedly improved on the Naf/DiG/Cu-Ag electrode, with values 1.5- and 5.2-fold greater than those of Cu-Ag and Cu electrodes, respectively (Fig. 6d). Furthermore, we conducted SEM analysis to investigate the surface reconstruction or degradation of Cu and Cu-Ag (Fig. S17). Previous studies have reported that Cu dissolution and redeposition readily occur during CO_2_RR, leading to surface reconstruction [40]. Consistent with this, the redeposited nanoparticles were observed on the electrode surface after CO_2_RR. Moreover, these phenomena are consistently observed on Naf/DiG/Cu and Naf/DiG/Cu-Ag. These observations indicate that the hetero-solvent microenvironment does not induce any additional or anomalous structural degradation beyond the intrinsic reconstruction inherent to CO_2_RR on Cu-based catalysts.

Comparison with state-of-the-art MEA-based CO_2_RR systems for C_2_H_5_OH production under neutral conditions (Fig. 6e, f and Table S2) [35, 66–70] reveals that our system delivers the highest C_2_H_5_OH partial current density while requiring a relatively low cell voltage of only 3.6 V (Fig. 6e). This performance advantage originates from effectively confining the hetero-solvent within the microenvironment rather than introducing it into the bulk electrolyte, thereby avoiding the penalties due to viscosity and ionic conductivity that typically limit high-current density operation [22, 27, 28]. Concurrently, our strategy enables not only considerable but also highly selective C_2_H_5_OH production, achieving an ethanol-to-ethylene ratio of 2.6 while maintaining the highest C_2_H_5_OH partial current density among reported systems under neutral conditions (Fig. 6f). Moreover, the hetero-solvent microenvironment is readily applicable across a wide range of conditions, including highly alkaline electrolytes (Fig. S18), different ionomer protective layers (Figs. S19 and S20), and different gas diffusion electrode (GDE) substrates (Figs. S21 and S22), underscoring the broad scalability of hetero-solvent strategy. Additionally, we performed the CO_2_RR measurement at varying KHCO_3_ concentrations. Previous studies have established that cation concentration plays a critical role in promoting C_2+_ production by stabilizing key CO_2_RR intermediates [71]. Accordingly, the C_2_/C_1_ selectivity decreases at lower KHCO_3_ concentrations for both Naf/Cu and Naf/DiG/Cu electrodes (Fig. S23). However, the effects of the DiG layer, suppressing HER and improving EtOH selectivity, remain consistently evident across all KHCO_3_ concentrations (Figs. S24 and S25). These results demonstrate that the DiG-based hetero-solvent effectively suppresses the competing HER and steers the reaction pathway toward ethanol production, largely independent of cation concentration.

In addition, to examine the stability of hetero-solvent microenvironment, long-term operation was conducted under 150 and 250 mA cm^–2^, as shown in Figs. 6g and S26, S27. A polytetrafluoroethylene (PTFE) substrate was employed to enhance the stability of the GDE, while humidified CO_2_ was supplied by passing it through a water bath maintained at 60 °C to mitigate salt accumulation [72]. The MEA with the Naf/DiG/Cu exhibited stable operation for 100 h. HER and C_2_H_4_ production were effectively suppressed, while C_2_H_5_OH production increased in Naf/DiG/Cu compared to bare Cu (Fig. S28), demonstrating practical applicability of our approach. Moreover, XPS and XRD analysis on used samples reveals negligible changes in the chemical states and crystalline structure of Naf/DiG/Cu compared to pristine Cu, suggesting negligible interaction between DiG and Cu during CO_2_RR (Figs. S29 and S30). Additionally, NMR analysis was performed to examine the possible dissolution of DiG during long-term CO_2_RR. During 100 h of CO_2_RR, the anolyte and cold trap were collected with interval of 12 h and subjected to NMR analysis. As shown in Fig. S31, no peaks corresponding to DiG were detected, suggesting negligible dissolution of DiG into the anolyte or cold trap during prolonged operation under 150 and 250 mA cm^–2^. After long-term CO_2_RR, the used membrane/electrode assembly (Sustainion/Naf/DiG/Cu/PTFE) was deliberately disassembled, and immersed in a water bath and subsequently analyzed by NMR (Fig. S32). A characteristic peak corresponding to DiG appeared at 3.2 ppm, confirming that DiG remained intact and confined within the catalyst microenvironment after 100 h of operation on the electrode. Consequently, these results demonstrate that the hetero-solvent microenvironment effectively maintains its role as a regulator of the hydrogen-bonding network of interfacial water, resulting in stable and efficient CO_2_-to-ethanol conversion.

## Conclusions

This work proposes a hetero-solvent microenvironment that modulates the hydrogen-bonding network of interfacial water to suppress HER and promote CO_2_-to-ethanol formation. Physicochemical analyses confirmed that the intrinsic properties of the Cu catalyst remained unchanged in the presence of Nafion and DiG, underscoring that the observed effects originated from modification of microenvironment rather than that of Cu catalyst. The Cu electrode with hetero-solvent microenvironment (Naf/DiG/Cu) exhibited significantly suppressed HER during CO_2_RR under neutral condition. Independent HER studies conducted under neutral and alkaline conditions revealed that hetero-solvent microenvironment plays a critical role in suppressing the Volmer step associated with water dissociation. *In situ* SEIRAS measurements further unveiled that the hetero-solvent strengthens the hydrogen-bonding network of interfacial water, thereby allowing us to establish a mechanistic link between HER activity and the hydrogen-bonding network of interfacial water within hetero-solvent microenvironment. Additionally, DFT calculations elucidated that the strengthened hydrogen-bonding network within the hetero-solvent microenvironment preferentially promotes the LH pathway over the ER pathway, thereby enabling selective ethanol production. Remarkably, the modulated hydrogen-bonding network of interfacial water enables selective production of C_2_H_5_OH. The Naf/DiG/Cu electrode achieved the highest C_2_H_5_OH partial current density of 89.5 mA cm^−2^, exceeding Naf/Cu and Cu by 2.6- and 5.3-fold, respectively. Isotope labeling experiments further support a correlation between hydrogen-bonding network of interfacial water and C_2_H_4_/C_2_H_5_OH selectivity. Increasing D_2_O content strengthens the hydrogen-bonding network, suppressing solvent hydrogenation, thereby leading to lower C_2_H_4_ selectivity. Consequently, the Naf/DiG/Cu electrode achieves superior C_2_H_5_OH selectivity due to restricted solvent hydrogenation that leads to C_2_H_4_ formation. Finally, the strategy of hetero-solvent microenvironment can be easily translated to other catalysts, as it does not rely on modification of the catalyst. The Naf/DiG/Cu-Ag electrode delivered a C_2_H_5_OH partial current density of 184.2 mA cm^−2^ at a cell voltage of 3.6 V, outperforming previous records from MEA-based CO_2_RR under neutral conditions. These findings establish interfacial water control *via* hetero-solvent microenvironment as an effective route to boost ethanol formation in CO_2_RR. In addition, the strategy of hetero-solvent microenvironment can offer a versatile and scalable design principle applicable to other electrochemical processes where interfacial water management is critical, such as ammonia synthesis, water electrolysis, and organic electrosynthesis in aqueous systems. 

## Supplementary Information

Below is the link to the electronic supplementary material.

Supplementary file1 (DOCX 43613 kb)

## References

[1] C. Wang, Z. Lv, X. Feng, W. Yang, B. Wang, Recent advances in electrochemical CO_2_-to-multicarbon conversion: from fundamentals to industrialization. Adv. Energy Mater. **13**(47), 2302382 (2023). 10.1002/aenm.202302382

[2] Y. Hori, K. Kikuchi, S. Suzuki, Production of co and CH_4_ in electrochemical reduction of CO_2_ at metal electrodes in aqueous hydrogencarbonate solution. Chem. Lett. **14**(11), 1695–1698 (1985). 10.1246/cl.1985.1695

[3] W. Liu, Z. Lv, X. Li, C. Wang, C. Tian et al., Nitrogen-rich porous-conjugated framework for efficient capture and electroreduction of simulated flue gas in acidic electrolyte. J. Am. Chem. Soc. **147**(27), 24023–24031 (2025). 10.1021/jacs.5c07519 40577593 10.1021/jacs.5c07519

[4] C. Kim, J.C. Bui, X. Luo, J.K. Cooper, A. Kusoglu et al., Tailored catalyst microenvironments for CO_2_ electroreduction to multicarbon products on copper using bilayer ionomer coatings. Nat. Energy **6**(11), 1026–1034 (2021). 10.1038/s41560-021-00920-8

[5] Y. Wang, J. Zhang, J. Zhao, Y. Wei, S. Chen et al., Strong hydrogen-bonded interfacial water inhibiting hydrogen evolution kinetics to promote electrochemical CO_2_ reduction to C_2+_. ACS Catal. **14**(5), 3457–3465 (2024). 10.1021/acscatal.3c05880

[6] L. Zhang, Z. Wei, S. Thanneeru, M. Meng, M. Kruzyk et al., A polymer solution to prevent nanoclustering and improve the selectivity of metal nanoparticles for electrocatalytic CO_2_ reduction. Angew. Chem. Int. Ed. **58**(44), 15834–15840 (2019). 10.1002/anie.201909069 10.1002/anie.201909069 31468668

[7] D. Zeng, C. Li, W. Wang, L. Zhang, Y. Zhang et al., Insights into the hydrophobic surface promoting electrochemical CO_2_ reduction to ethylene. Chem. Eng. J. **461**, 142133 (2023). 10.1016/j.cej.2023.142133

[8] Y.-J. Ko, C. Lim, J. Jin, M.G. Kim, J.Y. Lee et al., Extrinsic hydrophobicity-controlled silver nanoparticles as efficient and stable catalysts for CO_2_ electrolysis. Nat. Commun. **15**, 3356 (2024). 10.1038/s41467-024-47490-3 38637502 10.1038/s41467-024-47490-3 PMC11026478

[9] Z. Xing, L. Hu, D.S. Ripatti, X. Hu, X. Feng, Enhancing carbon dioxide gas-diffusion electrolysis by creating a hydrophobic catalyst microenvironment. Nat. Commun. **12**, 136 (2021). 10.1038/s41467-020-20397-5 33420043 10.1038/s41467-020-20397-5 PMC7794506

[10] L. Zhou, C. Li, J.-J. Lv, W. Wang, S. Zhu et al., Synergistic regulation of hydrophobicity and basicity for copper hydroxide-derived copper to promote the CO_2_ electroreduction reaction. Carbon Energy **5**(6), e328 (2023). 10.1002/cey2.328

[11] T.H.M. Pham, J. Zhang, M. Li, T.-H. Shen, Y. Ko et al., Enhanced electrocatalytic CO_2_ reduction to C_2+_ products by adjusting the local reaction environment with polymer binders. Adv. Energy Mater. **12**(9), 2270034 (2022). 10.1002/aenm.202270034

[12] M. Khalil, G.T.M. Kadja, F.A.A. Nugroho, L.G. Sutanto, P.K. Jiwanti et al., Suppressing the competing hydrogen evolution reaction in CO_2_ electroreduction: a review. Renew. Sustain. Energy Rev. **206**, 114869 (2024). 10.1016/j.rser.2024.114869

[13] C.N. Sun, Y.B. Qu, Z.L. Wang, J. Qing, Hydrogen spillover in alkaline solutions for effective nitrogen fixation. Chem. Eng. J. **471**, 144589 (2023). 10.1016/j.cej.2023.144589

[14] W. Liu, Z. Lv, C. Wang, C. Sun, C. Tian et al., Industrial-level modulation of catalyst-electrolyte microenvironment for electrocatalytic CO_2_ reduction: challenges and advancements. Adv. Energy Mater. **14**(44), 2402942 (2024). 10.1002/aenm.202402942

[15] Z. Chen, X. Duan, W. Wei, S. Wang, B.-J. Ni, Recent advances in transition metal-based electrocatalysts for alkaline hydrogen evolution. J. Mater. Chem. A **7**(25), 14971–15005 (2019). 10.1039/c9ta03220g

[16] Y. Zheng, Y. Jiao, A. Vasileff, S.-Z. Qiao, The hydrogen evolution reaction in alkaline solution: from theory, single crystal models, to practical electrocatalysts. Angew. Chem. Int. Ed. **57**(26), 7568–7579 (2018). 10.1002/anie.201710556 10.1002/anie.201710556 29194903

[17] X. Bai, C. Chen, X. Zhao, Y. Zhang, Y. Zheng et al., Accelerating the reaction kinetics of CO_2_ reduction to multi-carbon products by synergistic effect between cation and aprotic solvent on copper electrodes. Angew. Chem. Int. Ed. **63**(9), e202317512 (2024). 10.1002/anie.202317512 10.1002/anie.202317512 38168478

[18] Q. Wen, J. Duan, W. Wang, D. Huang, Y. Liu et al., Engineering a local free water enriched microenvironment for surpassing platinum hydrogen evolution activity. Angew. Chem. Int. Ed. **61**(35), e202206077 (2022). 10.1002/anie.202206077 10.1002/anie.202206077 35730919

[19] Y.-H. Wang, S. Zheng, W.-M. Yang, R.-Y. Zhou, Q.-F. He et al., *In situ* Raman spectroscopy reveals the structure and dissociation of interfacial water. Nature **600**(7887), 81–85 (2021). 10.1038/s41586-021-04068-z 34853456 10.1038/s41586-021-04068-z

[20] L.-F. Shen, B.-A. Lu, Y.-Y. Li, J. Liu, Z.-C. Huang-fu et al., Interfacial structure of water as a new descriptor of the hydrogen evolution reaction. Angew. Chem. Int. Ed. **59**(50), 22397–22402 (2020). 10.1002/anie.202007567 10.1002/anie.202007567 32893447

[21] Y. Zhao, J. Wang, X. Zha, X. Sheng, L. Dong et al., A cosolvent electrolyte boosting electrochemical alkynol semihydrogenation. J. Am. Chem. Soc. **147**(2), 1938–1947 (2025). 10.1021/jacs.4c14773 39745011 10.1021/jacs.4c14773

[22] S. Li, C.N. University, C.N. University, R. Shi et al., Structure and dissociation of water at the electrode–solution interface studied by *in situ* vibrational spectroscopic techniques. Anal. Chem. **97**(20), 10535–10549 (2025). 10.1021/acs.analchem.5c01651 40359500 10.1021/acs.analchem.5c01651

[23] N. Mohandas, T.N. Narayanan, A. Cuesta, Tailoring the interfacial water structure by electrolyte engineering for selective electrocatalytic reduction of carbon dioxide. ACS Catal. **13**(13), 8384–8393 (2023). 10.1021/acscatal.3c01223

[24] C. Zhu, Y. Han, L. Luo, L. Yan, B. Xiang et al., Dual modulation of electrolyte inner solvent structure and anode interface for high performance alkaline Al-air battery. Chem. Eng. J. **496**, 153814 (2024). 10.1016/j.cej.2024.153814

[25] L. Zhou, S. Tian, X. Du, T. Liu, H. Zhang et al., Suppressing hydrogen evolution in aqueous lithium-ion batteries with double-site hydrogen bonding. ACS Energy Lett. **8**(1), 40–47 (2023). 10.1021/acsenergylett.2c01993

[26] K. Xiao, L. Yang, M. Peng, X. Jiang, T. Hu et al., Unlocking the effect of chain length and terminal group on ethylene glycol ether family toward advanced aqueous electrolytes. Small **20**(12), 2306808 (2024). 10.1002/smll.202306808 10.1002/smll.202306808 37946662

[27] A.K. Sihag, F. Altmann et al., Using sorbitol as electrolyte additive to control interfacial environments in electrochemical CO_2_ reduction on silver. ACS Catal. **15**(19), 16643–16652 (2025). 10.1021/acscatal.5c04382 41063803 10.1021/acscatal.5c04382 PMC12501930

[28] Y. Guo, J. Gu, R. Zhang, S. Zhang, Z. Li et al., Molecular crowding effect in aqueous electrolytes to suppress hydrogen reduction reaction and enhance electrochemical nitrogen reduction. Adv. Energy Mater. **11**(36), 2101699 (2021). 10.1002/aenm.202101699

[29] J. Zhang, C. Zhang, M. Wang, Y. Mao, B. Wu et al., Isotopic labelling of water reveals the hydrogen transfer route in electrochemical CO_2_ reduction. Nat. Chem. **17**(3), 334–343 (2025). 10.1038/s41557-024-01721-8 39915658 10.1038/s41557-024-01721-8

[30] Y. Ouyang, L. Shi, X. Bai, C. Ling, Q. Li et al., Selectivity of electrochemical CO_2_ reduction toward ethanol and ethylene: the key role of surface-active hydrogen. ACS Catal. **13**(23), 15448–15456 (2023). 10.1021/acscatal.3c03797

[31] Y.C. Li, Z. Wang, T. Yuan, D.-H. Nam, M. Luo et al., Binding site diversity promotes CO_2_ electroreduction to ethanol. J. Am. Chem. Soc. **141**(21), 8584–8591 (2019). 10.1021/jacs.9b02945 31067857 10.1021/jacs.9b02945

[32] Z. Zhang, L. Bian, H. Tian, Y. Liu, Y. Bando et al., Tailoring the surface and interface structures of copper-based catalysts for electrochemical reduction of CO_2_ to ethylene and ethanol. Small **18**(18), 2107450 (2022). 10.1002/smll.202107450 10.1002/smll.202107450 35128790

[33] D. Shekhawat, J.J. Spivey, D.A. Berry, Fuel cells: Technologies for fuel processing. (Elsevier, 2011). 10.1016/B978-0-444-53563-4.10016-1

[34] M. Prashanthi, R. Sundaram, Integrated waste management in india: status and future prospects for environmental sustainability (Springer International Publishing, 2016), 10.1007/978-3-319-27228-3

[35] T.-U. Wi, R. University, Z.H. Levell, S. Hao et al., Selective and stable ethanol synthesis *via* electrochemical CO_2_ reduction in a solid electrolyte reactor. ACS Energy Lett. **10**(2), 822–829 (2025). 10.1021/acsenergylett.4c03091

[36] C. Wang, Z. Lv, Y. Liu, R. Liu, C. Sun et al., Hydrogen-bonded organic framework supporting atomic Bi–N_2_O_2_Sites for high-efficiency electrocatalytic CO_2_Reduction. Angew. Chem. Int. Ed. **63**(22), e202404015 (2024). 10.1002/anie.202404015 10.1002/anie.202404015 38530039

[37] C. Wang, Z. Lv, Y. Liu, L. Dai, R. Liu et al., Asymmetric Cu–N_1_O_3_ sites coupling atop-type and bridge-type adsorbed *C_1_ for electrocatalytic CO_2_-to-C_2_ conversion. Angew. Chem. Int. Ed. **63**(44), e202411216 (2024). 10.1002/anie.202411216 10.1002/anie.202411216 39044263

[38] C. Zhu, L. Zhou, Z. Zhang, C. Yang, G. Shi et al., Dynamic restructuring of epitaxial Au–Cu biphasic interface for tandem CO_2_-to-C_2+_ alcohol conversion. Chem **8**(12), 3288–3301 (2022). 10.1016/j.chempr.2022.08.016

[39] S.H. Lee, J.E. Avilés Acosta, S. University et al., Structural transformation and degradation of Cu oxide nanocatalysts during electrochemical CO_2_ reduction. J. Am. Chem. Soc. **147**(8), 6536–6548 (2025). 10.1021/jacs.4c14720 39815387 10.1021/jacs.4c14720 PMC11869297

[40] I. Kim, G.-B. Lee, S. Kim, H.D. Jung, J.-Y. Kim et al., Unveiling the reconstruction of copper bimetallic catalysts during CO_2_ electroreduction. Nat. Catal. **8**(7), 697–713 (2025). 10.1038/s41929-025-01368-9

[41] Index. In: Advances in feedstock conversion technologies for alternative fuels and bioproducts. (Elsevier, 2019), pp. 373–390 10.1016/b978-0-12-817937-6.00034-5

[42] L.M. Palma, T.S. Almeida, A.R. de Andrade, Comparative study of catalyst effect on ethanol electrooxidation in alkaline medium: Pt- and Pd-based catalysts containing Sn and Ru. J. Electroanal. Chem. **878**, 114592 (2020). 10.1016/j.jelechem.2020.114592

[43] J. Campeggio, V. Volkov, M. Innocenti, W. Giurlani, C. Fontanesi et al., Ethanol electro-oxidation reaction on the Pd(111) surface in alkaline media: insights from quantum and molecular mechanics. Phys. Chem. Chem. Phys. **24**(20), 12569–12579 (2022). 10.1039/d2cp00909a 35579265 10.1039/d2cp00909a

[44] J.P. Guthrie, The aldol condensation of acetaldehyde: the equilibrium constant for the reaction and the rate constant for the hydroxide catalyzed RetroAldol reaction. Can. J. Chem. **52**(11), 2037–2040 (1974). 10.1139/v74-294

[45] A.W. Klaassen, C.G. Hill, Raman studies of aldol condensation reactions on sodium hydroxide-treated silica gel. J. Catal. **69**(2), 299–311 (1981). 10.1016/0021-9517(81)90167-6

[46] L. Zhang, T. Kang, J. Kang, X. Zhang, R. Zhang et al., Effects of electrolyte pH on the electro-osmotic characteristics in anthracite. ACS Omega **5**(45), 29257–29264 (2020). 10.1021/acsomega.0c04013 33225156 10.1021/acsomega.0c04013 PMC7676344

[47] M. Duss, R. Taylor, Predict distillation tray efficiency. Chem. Eng. Prog. **112**(6), 24–30 (2018) https://www.aiche.org/resources/publications/cep/2018/july/predict-distillation-tray-efficiency

[48] H.-F. Wang, Y.-G. Yan, S.-J. Huo, W.-B. Cai, Q.-J. Xu et al., Seeded growth fabrication of Cu-on-Si electrodes for *in situ* ATR-SEIRAS applications. Electrochim. Acta **52**(19), 5950–5957 (2007). 10.1016/j.electacta.2007.03.042

[49] P.E. Blöchl, Projector augmented-wave method. Phys. Rev. B **50**(24), 17953–17979 (1994). 10.1103/physrevb.50.17953 10.1103/physrevb.50.17953 9976227

[50] J.P. Perdew, K. Burke, M. Ernzerhof, Generalized gradient approximation made simple. Phys. Rev. Lett. **77**(18), 3865–3868 (1996). 10.1103/physrevlett.77.3865 10062328 10.1103/PhysRevLett.77.3865

[51] S. Grimme, Semiempirical GGA-type density functional constructed with a long-range dispersion correction. J. Comput. Chem. **27**(15), 1787–1799 (2006). 10.1002/jcc.20495 16955487 10.1002/jcc.20495

[52] S. Grimme, J. Antony, S. Ehrlich, H. Krieg, A consistent and accurate *ab initio* parametrization of density functional dispersion correction (DFT-D) for the 94 elements H-Pu. J. Chem. Phys. **132**(15), 154104 (2010). 10.1063/1.3382344 20423165 10.1063/1.3382344

[53] V. Wang, N. Xu, J.-C. Liu, G. Tang, W.-T. Geng, VASPKIT: a user-friendly interface facilitating high-throughput computing and analysis using VASP code. Comput. Phys. Commun. **267**, 108033 (2021). 10.1016/j.cpc.2021.108033

[54] Q.-C. Chen, W. Zhu, Y. Chen, H. An, S. Yang et al., High-asymmetry bipolar membrane electrode assemblies generate a superconcentration of cations and hydroxide at a catalyst surface. Energy Environ. Sci. **19**(5), 1530–1539 (2026). 10.1039/d5ee04672f

[55] R. Kas, K. Yang, D. Bohra, R. Kortlever, T. Burdyny et al., Electrochemical CO_2_ reduction on nanostructured metal electrodes: fact or defect? Chem. Sci. **11**(7), 1738–1749 (2020). 10.1039/c9sc05375a 34123269 10.1039/c9sc05375a PMC8150108

[56] V.G. Agarwal, S. Haussener, Quantifying mass transport limitations in a microfluidic CO_2_ electrolyzer with a gas diffusion cathode. Commun. Chem. **7**, 47 (2024). 10.1038/s42004-024-01122-5 38443453 10.1038/s42004-024-01122-5 PMC10914812

[57] F.P. García de Arquer, C.-T. Dinh, A. Ozden, J. Wicks, C. McCallum et al., CO_2_ electrolysis to multicarbon products at activities greater than 1 A cm^-2^. Science **367**(6478), 661–666 (2020). 10.1126/science.aay4217 32029623 10.1126/science.aay4217 PMC13531662

[58] A. Wuttig, M. Yaguchi, K. Motobayashi, M. Osawa, Y. Surendranath, Inhibited proton transfer enhances Au-catalyzed CO_2_-to-fuels selectivity. Proc. Natl. Acad. Sci. U.S.A. **113**(32), E4585–E4593 (2016). 10.1073/pnas.1602984113 27450088 10.1073/pnas.1602984113 PMC4987813

[59] T. Shinagawa, A.T. Garcia-Esparza, K. Takanabe, Insight on Tafel slopes from a microkinetic analysis of aqueous electrocatalysis for energy conversion. Sci. Rep. **5**, 13801 (2015). 10.1038/srep13801 26348156 10.1038/srep13801 PMC4642571

[60] T. Cheng, H. Xiao, W.A. Goddard III., Full atomistic reaction mechanism with kinetics for CO reduction on Cu(100) from *ab initio* molecular dynamics free-energy calculations at 298 K. Proc. Natl. Acad. Sci. U.S.A. **114**(8), 1795–1800 (2017). 10.1073/pnas.1612106114 28167767 10.1073/pnas.1612106114 PMC5338443

[61] X. Wang, Z. Wang, F.P. García de Arquer, C.-T. Dinh, A. Ozden et al., Efficient electrically powered CO_2_-to-ethanol *via* suppression of deoxygenation. Nat. Energy **5**, 478–486 (2020). 10.1038/s41560-020-0607-8

[62] F.J. Sarabia, P. Sebastián-Pascual, M.T.M. Koper, V. Climent, J.M. Feliu, Effect of the interfacial water structure on the hydrogen evolution reaction on Pt(111) modified with different nickel hydroxide coverages in alkaline media. ACS Appl. Mater. Interfaces **11**(1), 613–623 (2019). 10.1021/acsami.8b15003 30539624 10.1021/acsami.8b15003

[63] Y. Liang, F. Li, R.K. Miao, S. Hu, W. Ni et al., Efficient ethylene electrosynthesis through C–O cleavage promoted by water dissociation. Nat. Synth. **3**(9), 1104–1112 (2024). 10.1038/s44160-024-00568-8

[64] W. Su, W. Guo, Y. Fan, CuAg bimetallic catalysts derived from an Ag-anchored Cu-based metal–organic framework for CO_2_ electroreduction to ethanol. Chem. Eng. J. **477**, 147204 (2023). 10.1016/j.cej.2023.147204

[65] P. Luan, X. Dong, L. Liu, J. Xiao, P. Zhang et al., Selective electrosynthesis of ethanol *via* asymmetric C-C coupling in tandem CO_2_ reduction. ACS Catal. **14**(11), 8776–8785 (2024). 10.1021/acscatal.4c01579

[66] Z. Gu, H. Shen, Z. Chen, Y. Yang, C. Yang et al., Efficient electrocatalytic CO_2_ reduction to C_2+_ alcohols at defect-site-rich Cu surface. Joule **5**(2), 429–440 (2021). 10.1016/j.joule.2020.12.011

[67] R.K. Miao, Y. Xu, A. Ozden, A. Robb, C.P. O’Brien et al., Electroosmotic flow steers neutral products and enables concentrated ethanol electroproduction from CO_2_. Joule **5**(10), 2742–2753 (2021). 10.1016/j.joule.2021.08.013

[68] S.-J. Shin, H. Choi, S. Ringe, D.H. Won, H.-S. Oh et al., A unifying mechanism for cation effect modulating C_1_ and C_2_ productions from CO_2_ electroreduction. Nat. Commun. **13**, 5482 (2022). 10.1038/s41467-022-33199-8 36123326 10.1038/s41467-022-33199-8 PMC9485141

[69] T. Zhao, X. Zong, J. Liu, J. Chen, K. Xu et al., Functionalizing Cu nanoparticles with fluoric polymer to enhance C_2+_ product selectivity in membraned CO_2_ reduction. Appl. Catal. B Environ. **340**, 123281 (2024). 10.1016/j.apcatb.2023.123281

[70] F. Li, Y.C. Li, Z. Wang, J. Li, D.-H. Nam et al., Cooperative CO_2_-to-ethanol conversion *via* enriched intermediates at molecule–metal catalyst interfaces. Nat. Catal. **3**(1), 75–82 (2020). 10.1038/s41929-019-0383-7

[71] W. Li, Z. Yin, Z. Gao, G. Wang, Z. Li et al., Bifunctional ionomers for efficient co-electrolysis of CO_2_ and pure water towards ethylene production at industrial-scale current densities. Nat. Energy **7**(9), 835–843 (2022). 10.1038/s41560-022-01092-9

[72] D.G. Wheeler, B.A.W. Mowbray, A. Reyes, F. Habibzadeh, J. He et al., Quantification of water transport in a CO_2_ electrolyzer. Energy Environ. Sci. **13**(12), 5126–5134 (2020). 10.1039/d0ee02219e

