# Supplementary material for: Hetero-Solvent Microenvironment for Selective CO2 to Ethanol Electrolysis via Interfacial Water Control
Source: Nanomicro Lett. 2026 Jul 3;18:423. doi: 10.1007/s40820-026-02282-w (PMC13332080; doi:10.1007/s40820-026-02282-w)
Supplement: Supplementary file 1 — Supplementary file1 (DOCX 43613 kb) [file 40820_2026_2282_MOESM1_ESM.docx]

Supplementary Information for

**Hetero-Solvent Microenvironment for Selective CO_2_ to Ethanol Electrolysis *via* Interfacial Water Control**

Dohun Kim^1#^, Suyun Lee^1#^, Seeun Jung^2#^, Jaemin Kim^3^, Junsic Cho^2^, Dong Ki Lee^4,5^, Seoin Back^6,7,8*^, Chang Hyuck Choi^2*^, and Chanyeon Kim^1*^

^1^ Department of Energy Science and Engineering, Daegu Gyeongbuk Institute of Science & Technology (DGIST), Daegu 42988, Republic of Korea

^2^ Department of Chemistry, Pohang University of Science and Technology (POSTECH), Pohang 37673, Republic of Korea

^3^ Department of Chemical and Biomolecular Engineering, Sogang University, Seoul 04107, Republic of Korea

^4^ Clean Energy Research Center, Korea Institute of Science and Technology, Seoul, 02792 Republic of Korea

^5^ Department of Chemical and Biomolecular Engineering, Yonsei-KIST Convergence Research Institute, Yonsei University, Seoul, 03722 Republic of Korea

^6^ Department of Integrative Energy Engineering, Korea University, Seoul 02841, Republic of Korea

^7^ KU-KIST Graduate School of Converging Science and Technology, Korea University, 145 Anam-ro, Seongbuk-gu, Seoul, 02841, Republic of Korea

^8^ Institute for Multiscale Matter and Systems (IMMS), Ewha Womans University, Seoul 03760, Republic of Korea

^#^ Dohun Kim, Suyun Lee, and Seeun Jung Contributed equally to this work.

*Corresponding authors. E-mail: chanyeon@dgist.ac.kr (Chanyeon Kim); chchoi@postech.ac.kr (Chang Hyuck Choi); sback@korea.ac.kr (Seoin Back)

**Supplementary Figures and Tables**

**Table S1 |** The systematic comparison of candidate co-solvent

| Solvent | CO_2_ solubility [S1, S2] | Aprotic, H-bond accepting Characteristic [S3] | Electrochemical Stability [S4] | Boiling Point [S5] |
| --- | --- | --- | --- | --- |
| Water | ~34 mM | Protic, H-bond acceptor/donor |  | 100 ℃ |
| Dimethylformamide (DMF) | ~200 mM | Aprotic, H-bond acceptor | Unstable | 153 ℃ |
| Tetrahydrofuran  (THF) | ~200 mM | Aprotic, H-bond acceptor | Stable | 66 ℃ |
| Diglyme  (DiG) | ~160 mM | Aprotic, H-bond acceptor | Stable | 162 ℃ |

**
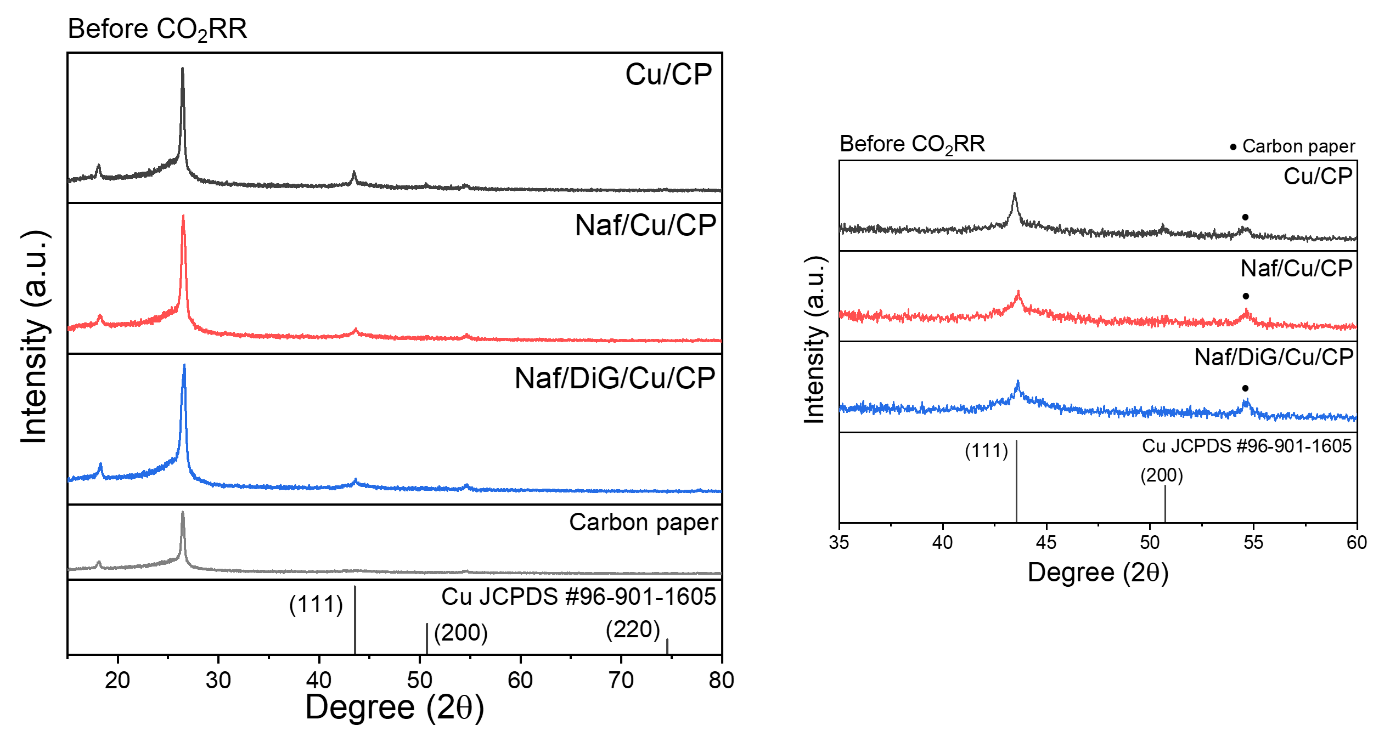
**

**Fig. S1 | XRD analysis before CO_2_RR.** XRD patterns of Cu, Naf/Cu, Naf/DiG/Cu on CP before CO_2_RR.


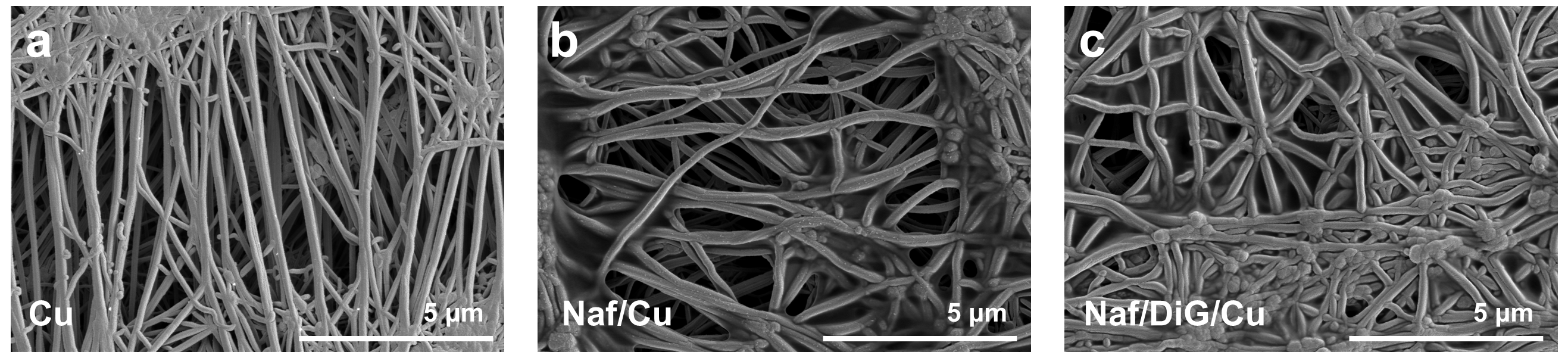


**Fig. S2 |** SEM analysis for (a) Cu, (b) Naf/Cu, and (c) Naf/DiG/Cu.


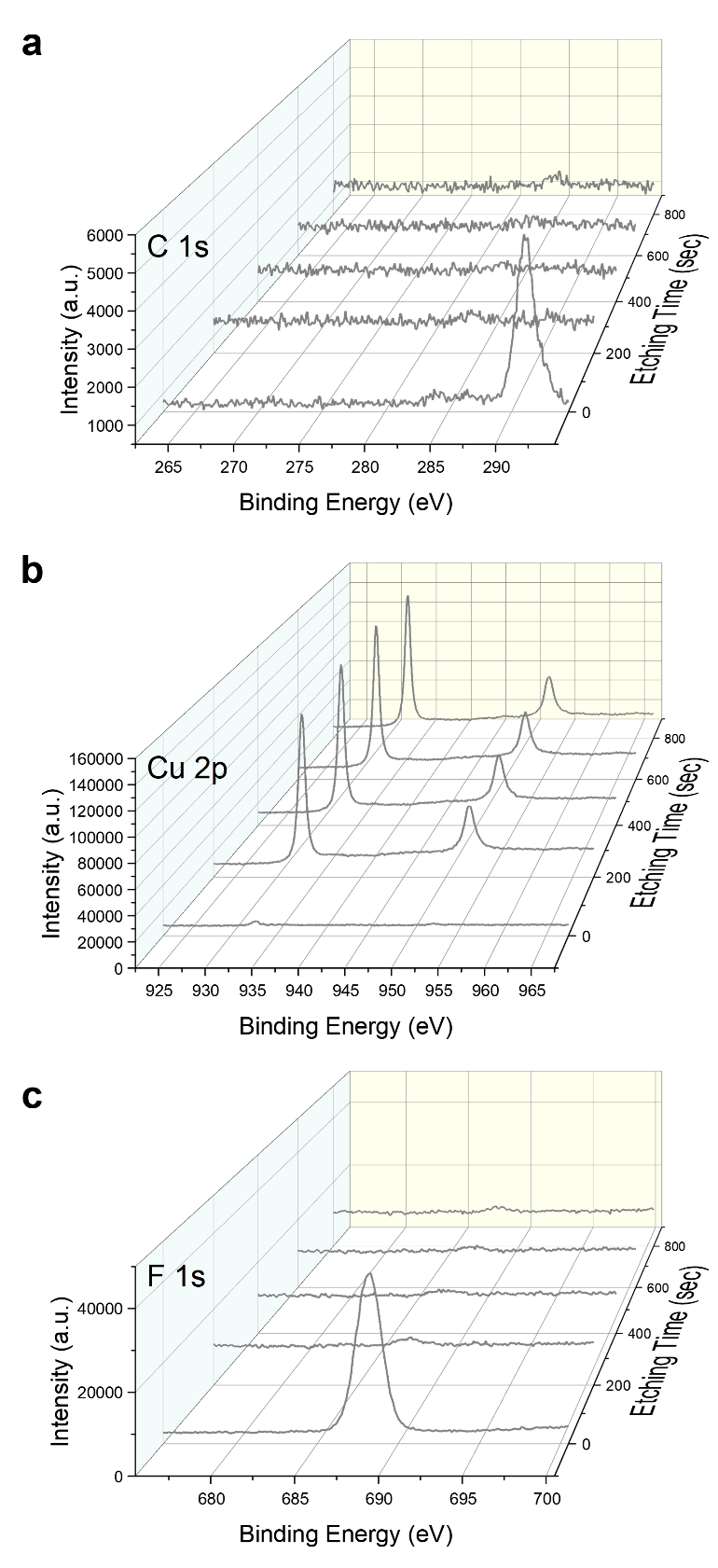


**Fig. S3 | XPS depth profile spectra of Naf/Cu. a-c,** XPS spectra of C 1s (**a**), Cu 2p (**b**), and F 1s (**c**) based on Ar etching time.


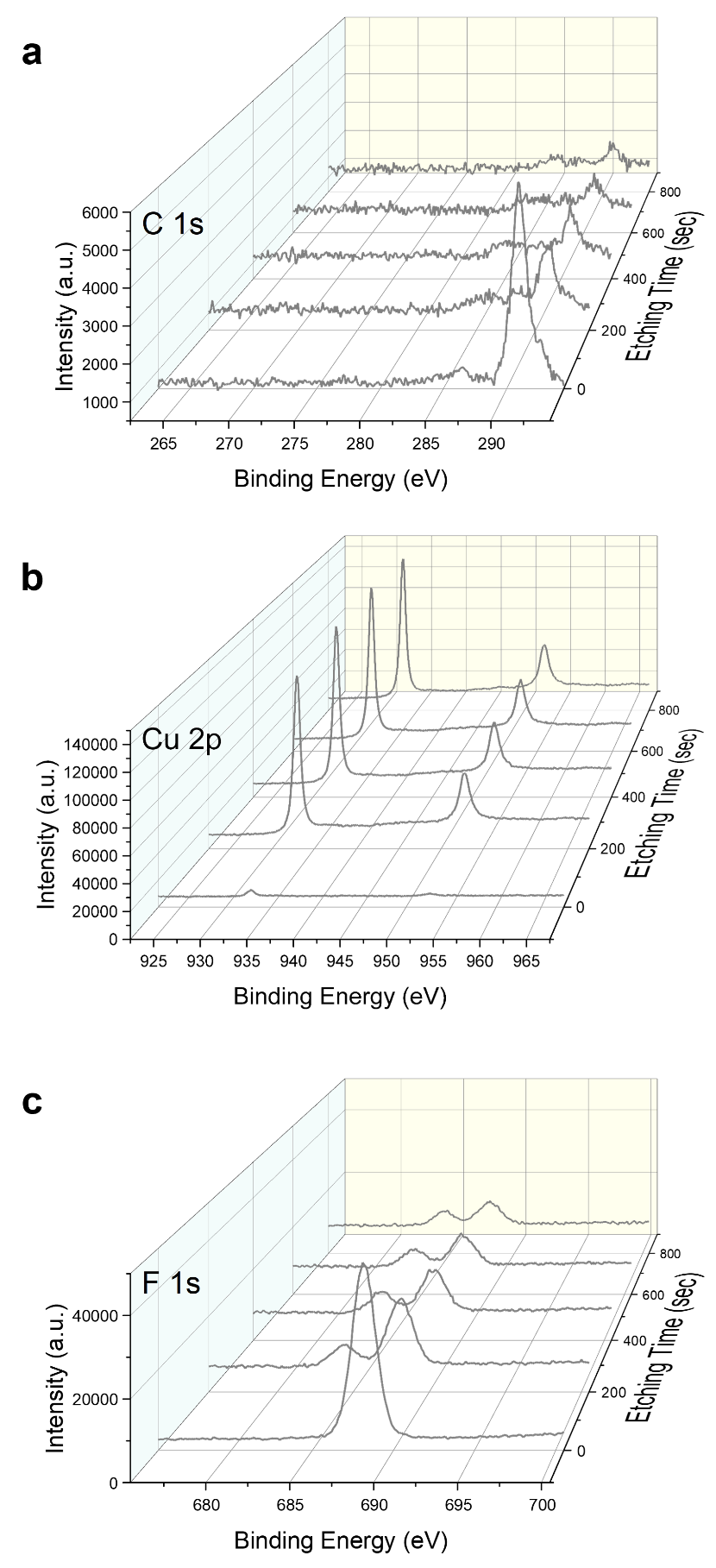


**Fig. S4 | XPS depth profile spectra of Naf/DiG/Cu. a-c,** XPS spectra of C 1s (**a**), Cu 2p (**b**), and F 1s (**c**) based on Ar etching time.


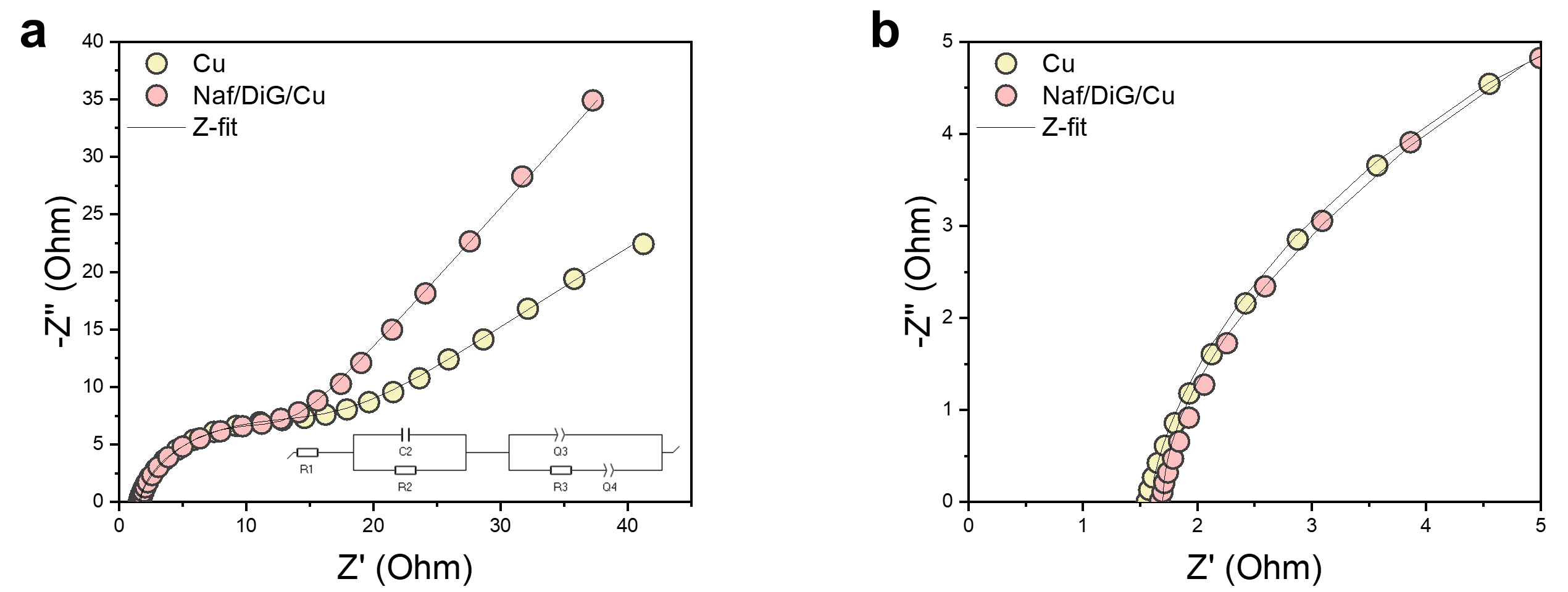


**Fig. S5 | EIS measurements for Cu and Naf/DiG/Cu.** (a) Nyquist plots over the full frequency range and (b) enlarged view of the high-frequency region.


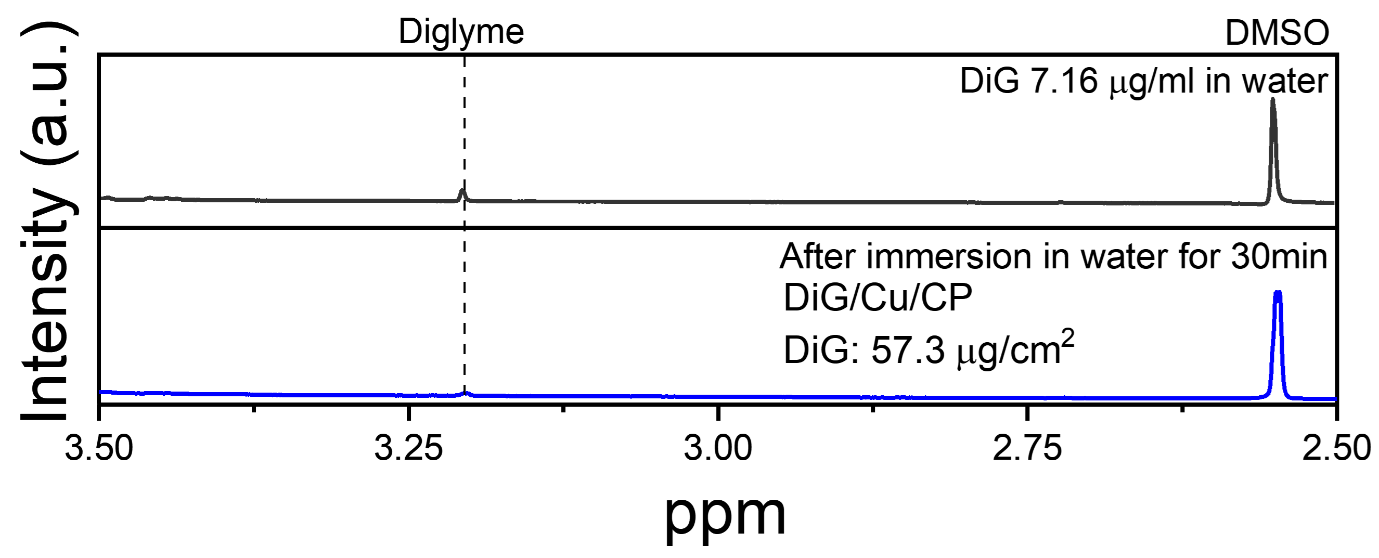


**Fig. S6 | NMR analysis to confirm the dissolution of Diglyme in water.** NMR spectra of DiG/Cu after immersion in water for 30 min.


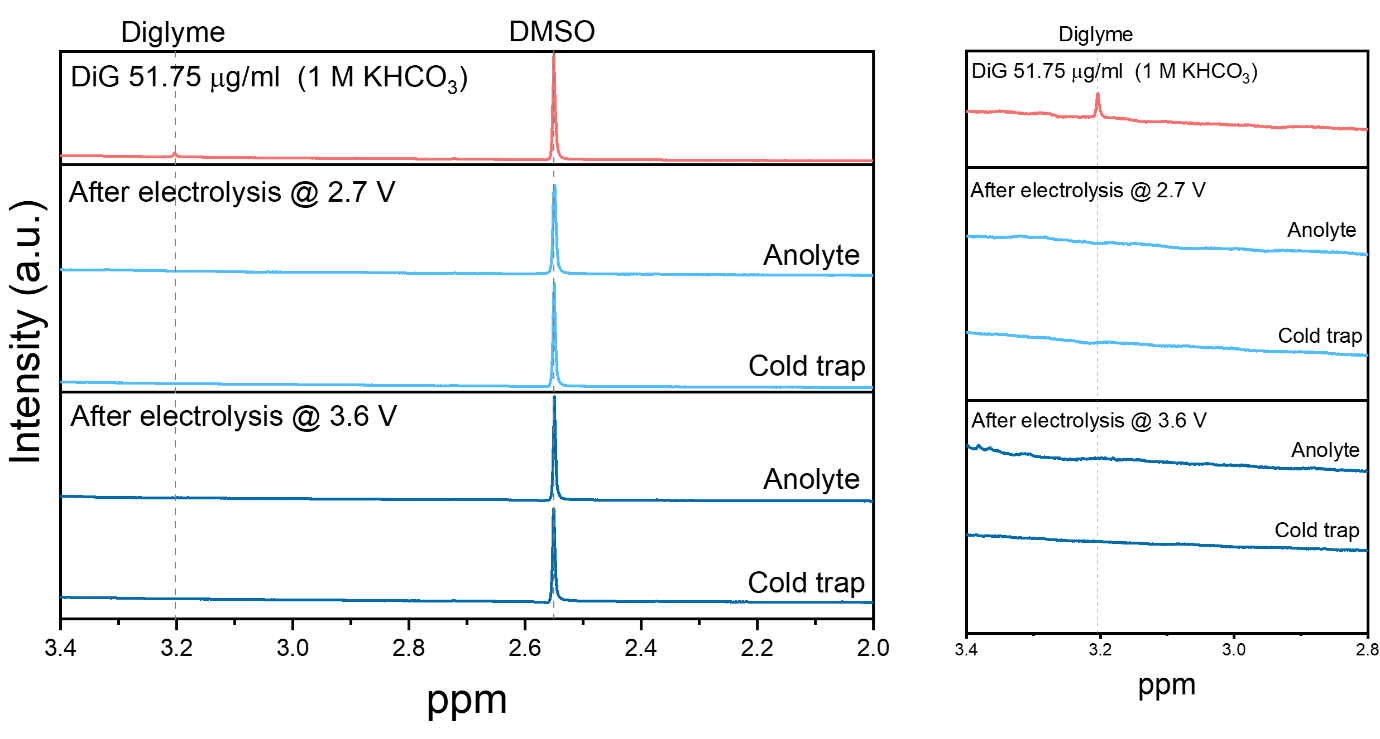


**Fig. S7 | NMR analysis to identify the Nafion protective effect on dissolution of Diglyme.** NMR spectra of anolyte and cold trap after electrolysis at 2.7 and 3.6 V using Naf/DiG/Cu.


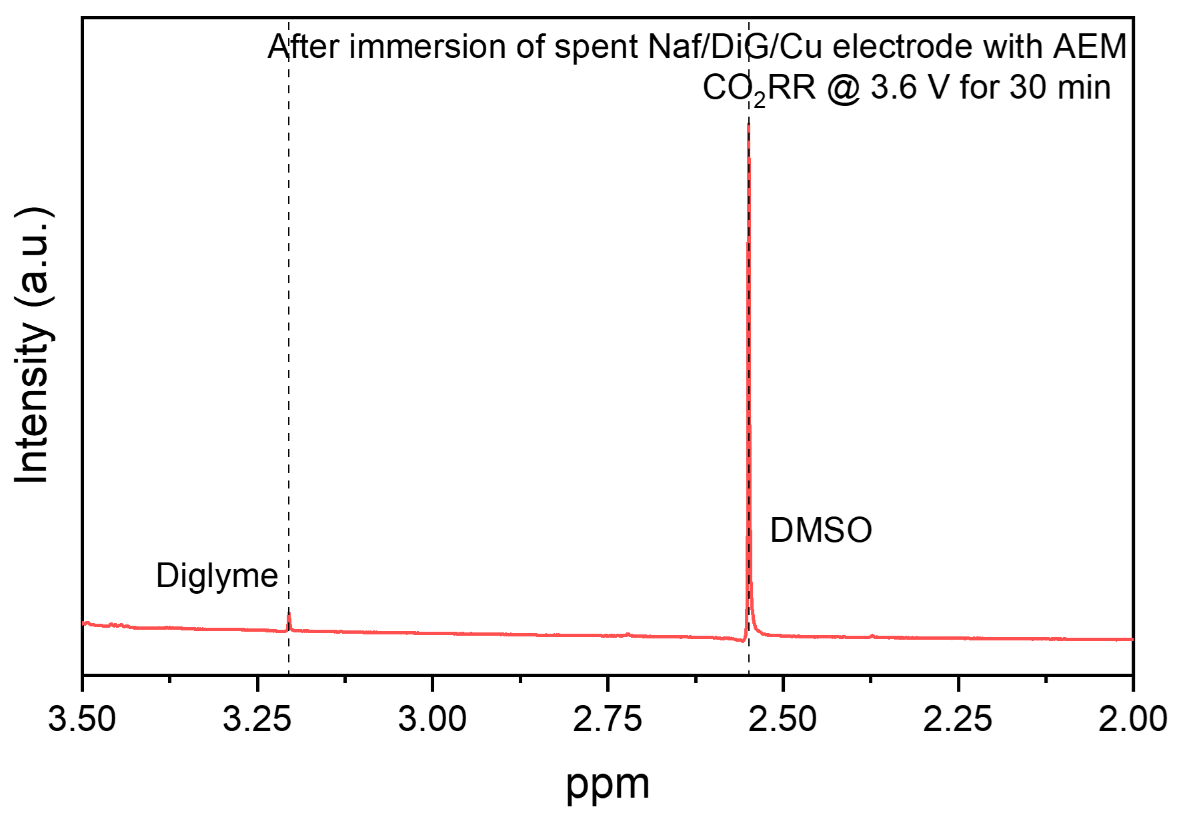


**Fig. S8 | NMR analysis to identify the Nafion protective effect on dissolution of Diglyme.** NMR spectra of the used MEA after CO_2_RR was deliberately torn apart and immersed in water.


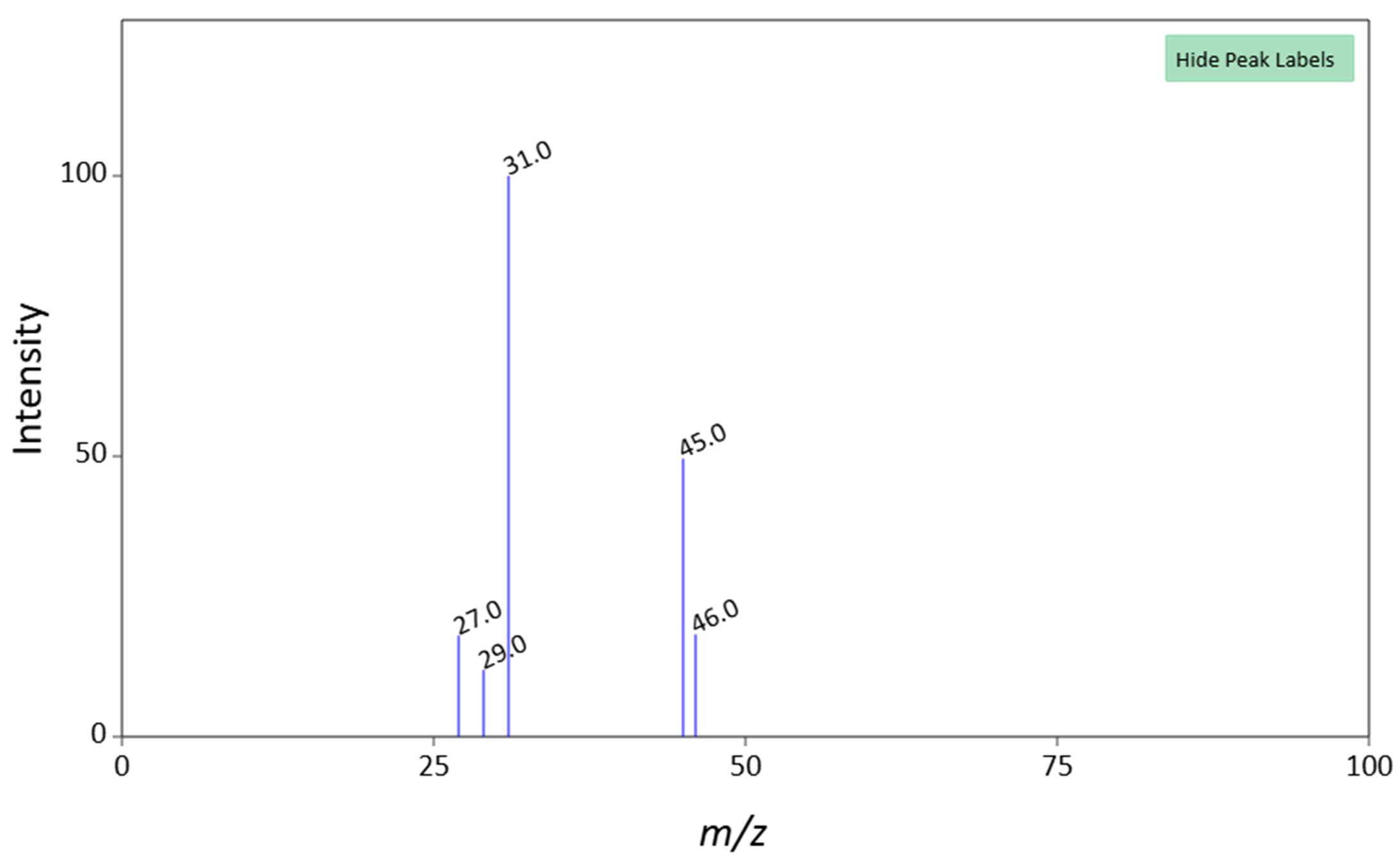


**Fig. S9 |** Candidate products derived from the decomposition of ethanol.

*
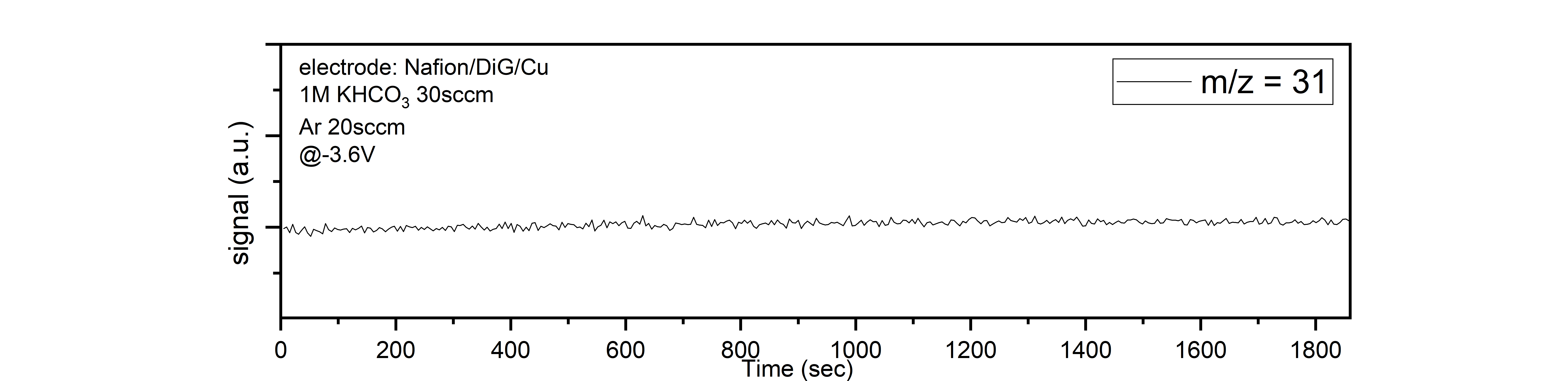
*

**Fig. S10 |** Online DEMS spectra for ethanol fragment under Ar flow at 3.6 V over 30 min.


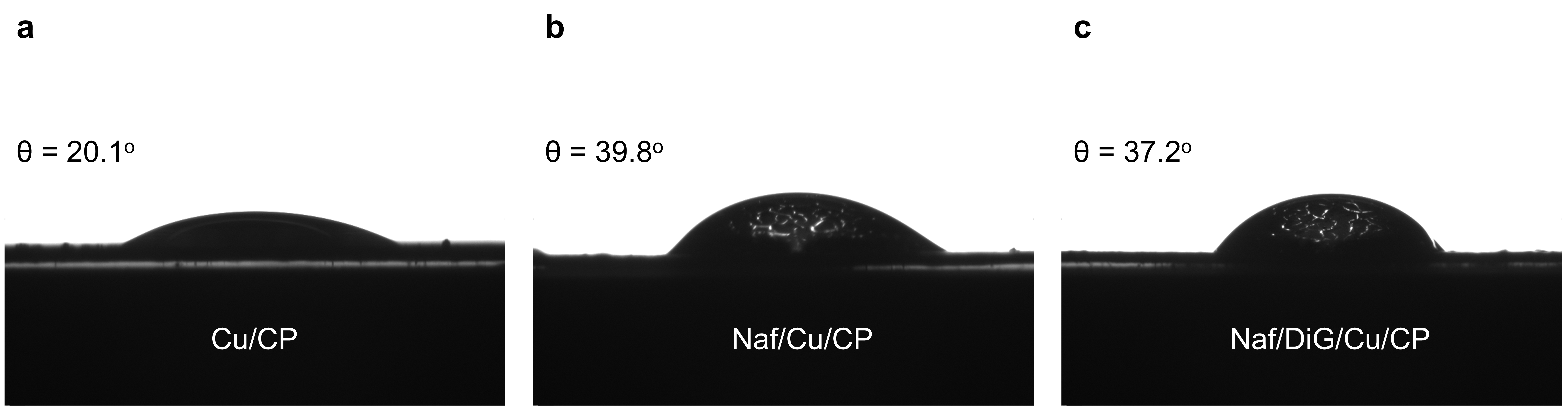


**Fig. S11 | Nafion effect on hydrophobicity.** Contact angle analysis for (a) Cu/CP, (b) Naf/Cu/CP, and (c) Naf/DiG/Cu/CP.


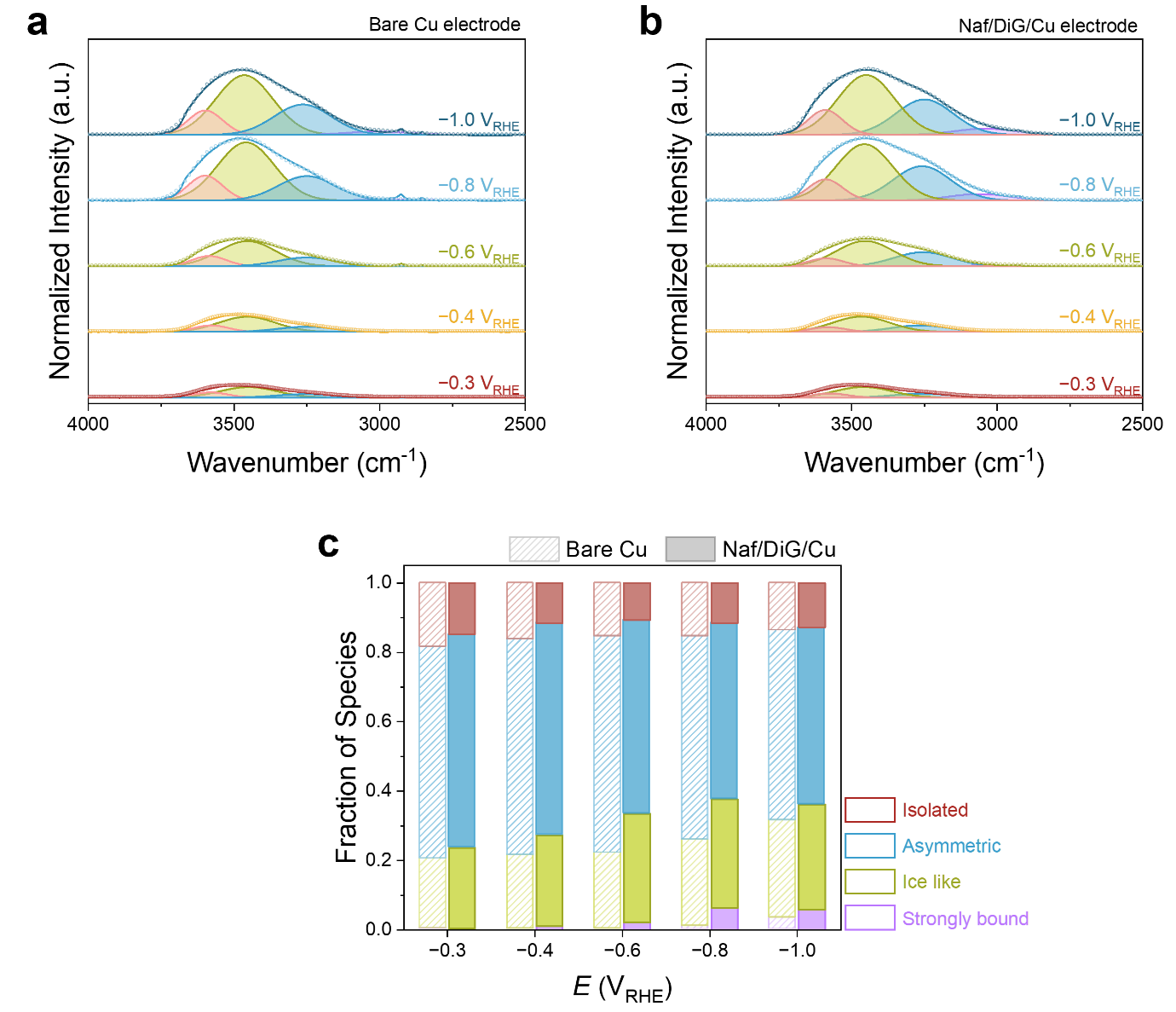


**Fig. S12 | *In situ* SEIRAS spectra. a-b,** *In situ* SEIRAS spectra of interfacial water during the CO_2_RR on Cu (**a**) and Naf/DiG/Cu (**b**). The spectra were deconvoluted into four components representing different hydrogen-bonding environments: strongly bound (purple), ice-like (green), asymmetric (blue), and isolated/free (red) water species [S6]. **c**, Fractions of the water species at various applied potentials. In all potential ranges, the Naf/DiG/Cu shows a higher contribution of ice-like water, and a corresponding lower contribution of asymmetric and isolated/free water compared to the bare Cu. This fitting result aligns well with the trend obtained from the center of mass frequency (ν_COM_). However, to avoid the potential ambiguities associated with multi-component fitting, we employed ν_COM_ for further discussion in the main text.


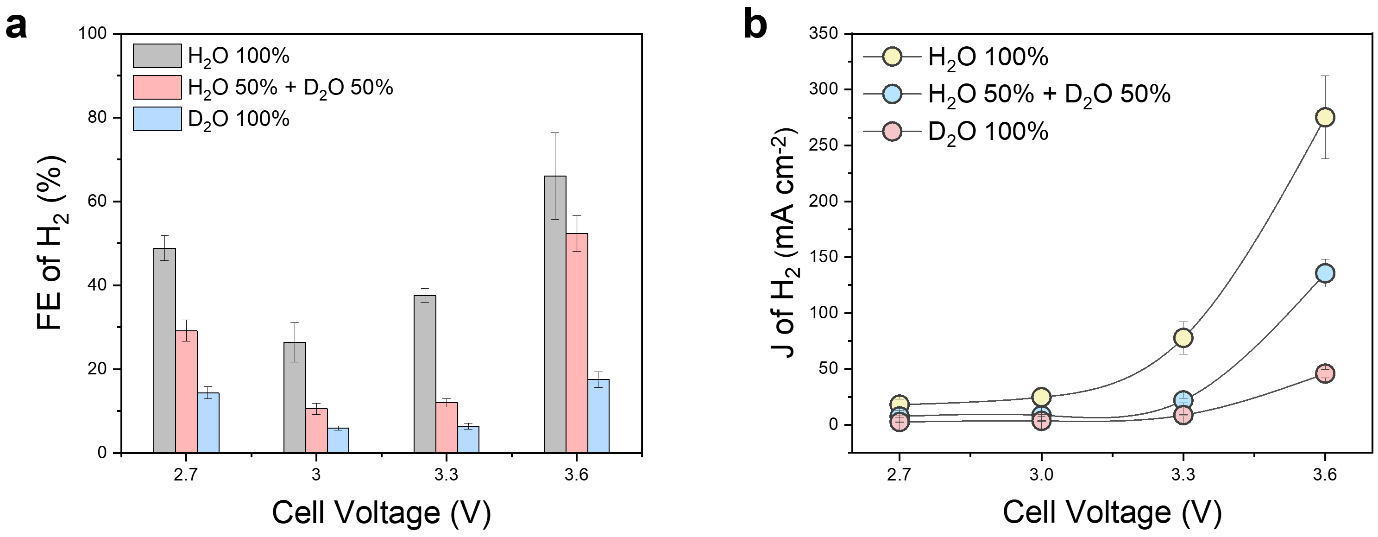


**Fig. S13 | Isotope analysis to investigate the correlation between water activity and CO_2_RR selectivity. a-b,** FE (**a**) and partial current density (**b**) of H_2_ depending on H_2_O/D_2_O ratio.


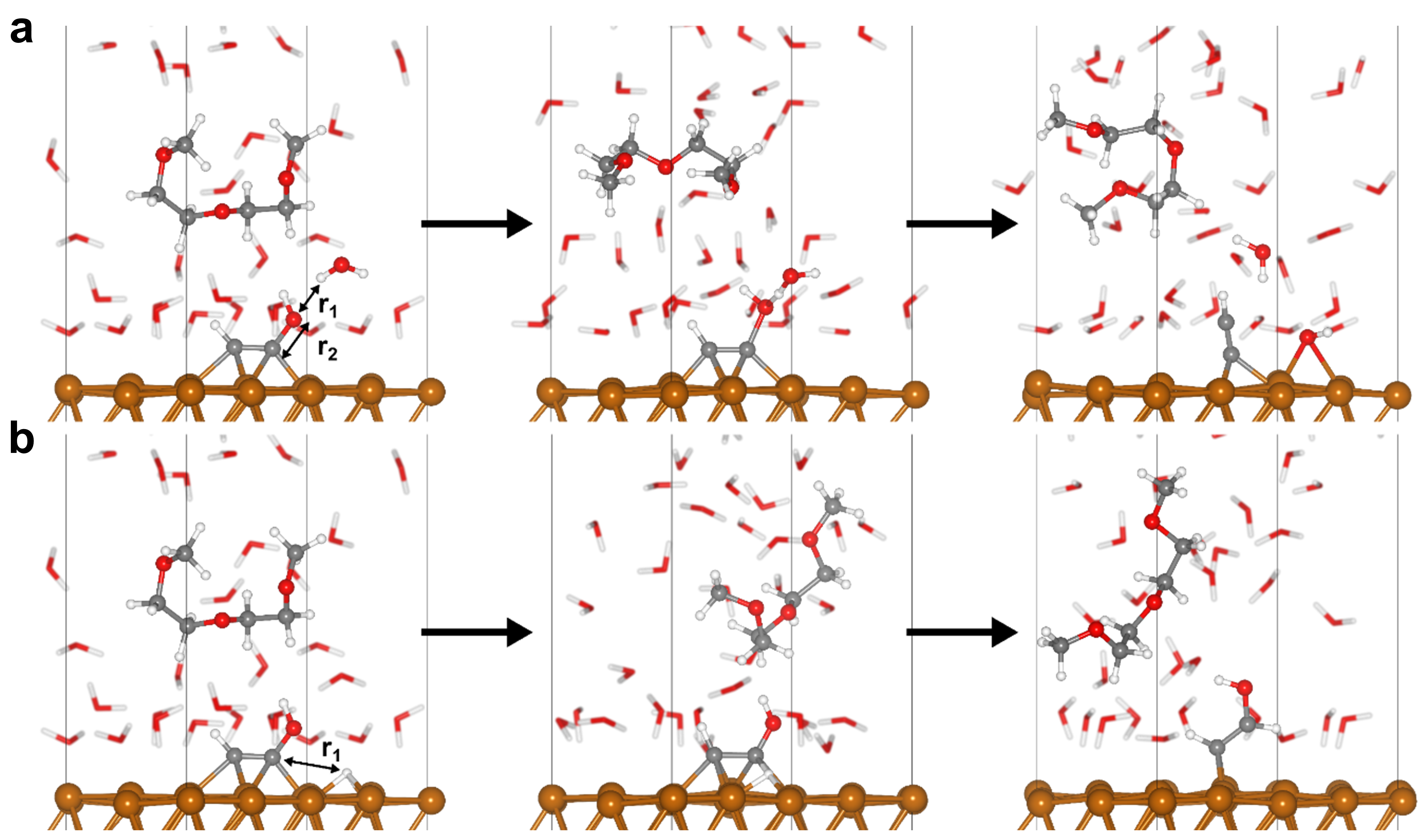


**Fig. S14 |** Representative structures from the slow-growth AIMD simulations of the (a) ER mechanism and (b) LH mechanism on the DiG/Cu surface. The DiG molecule and the atoms actively participating in the reaction are shown in ball-and-stick representation, while the background water molecules are depicted as faded sticks for clarity.


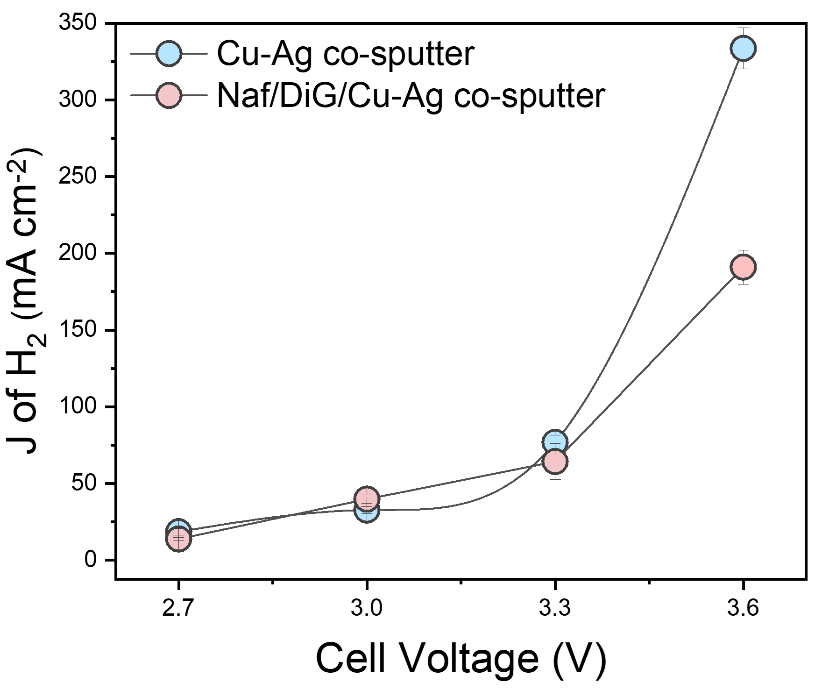


**Fig. S15 | Hetero-solvent effects on Cu-Ag electrode.** Partial current densities for H_2_ of Cu-Ag and Naf/DiG/Cu-Ag.


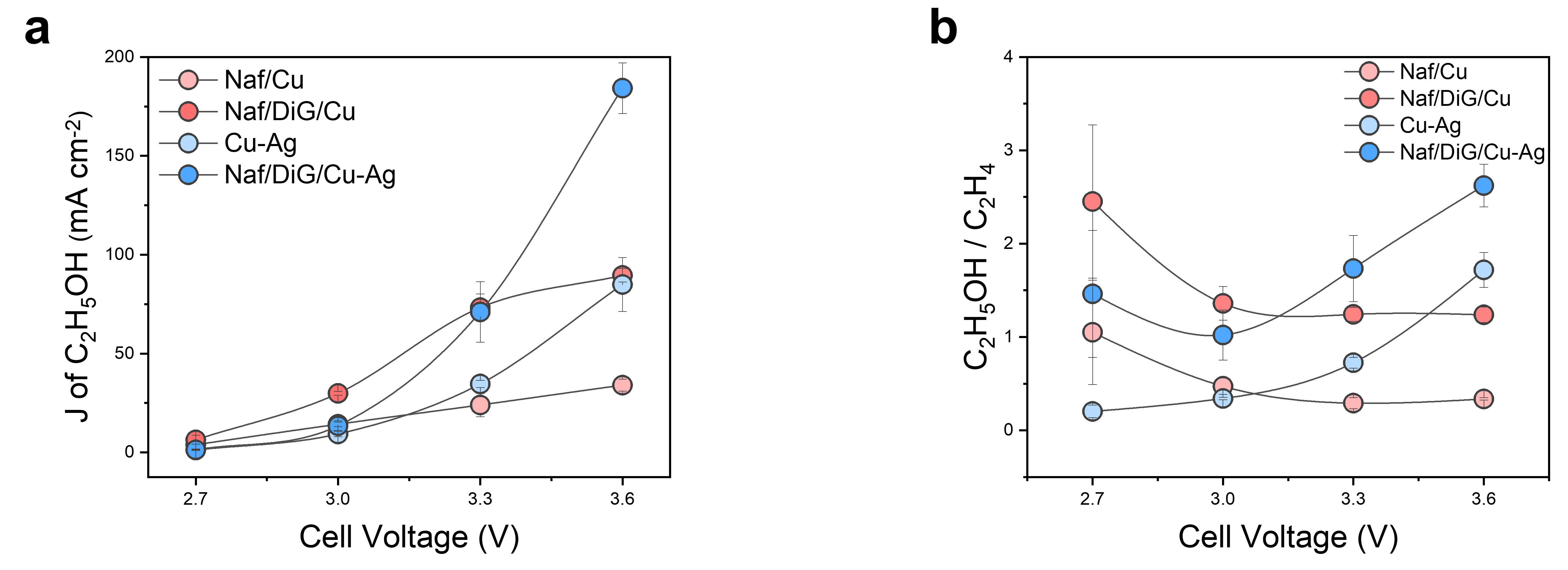


**Fig. S16 |** (a) Partial current density and (b) selectivity for ethanol production.


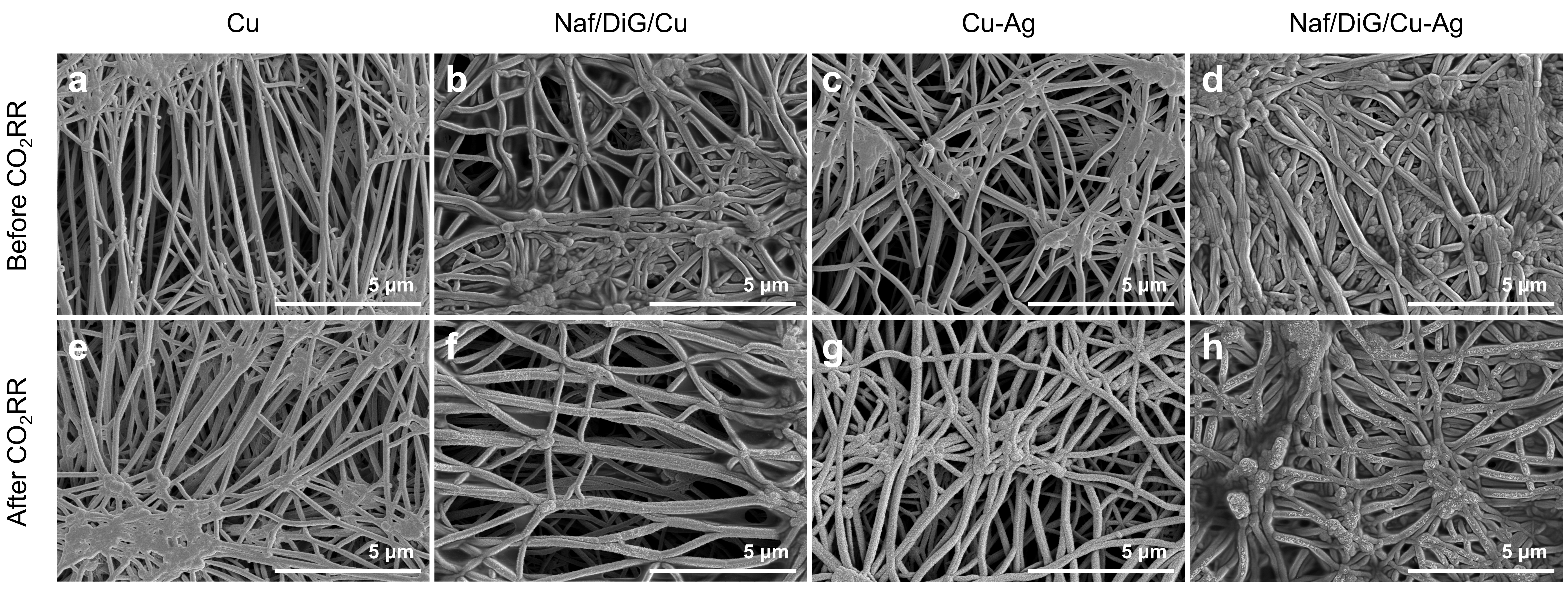


**Fig. S17 |** SEM images of Cu, Naf/DiG/Cu, Cu-Ag, and Naf/DiG/Cu-Ag before/after CO_2_RR.


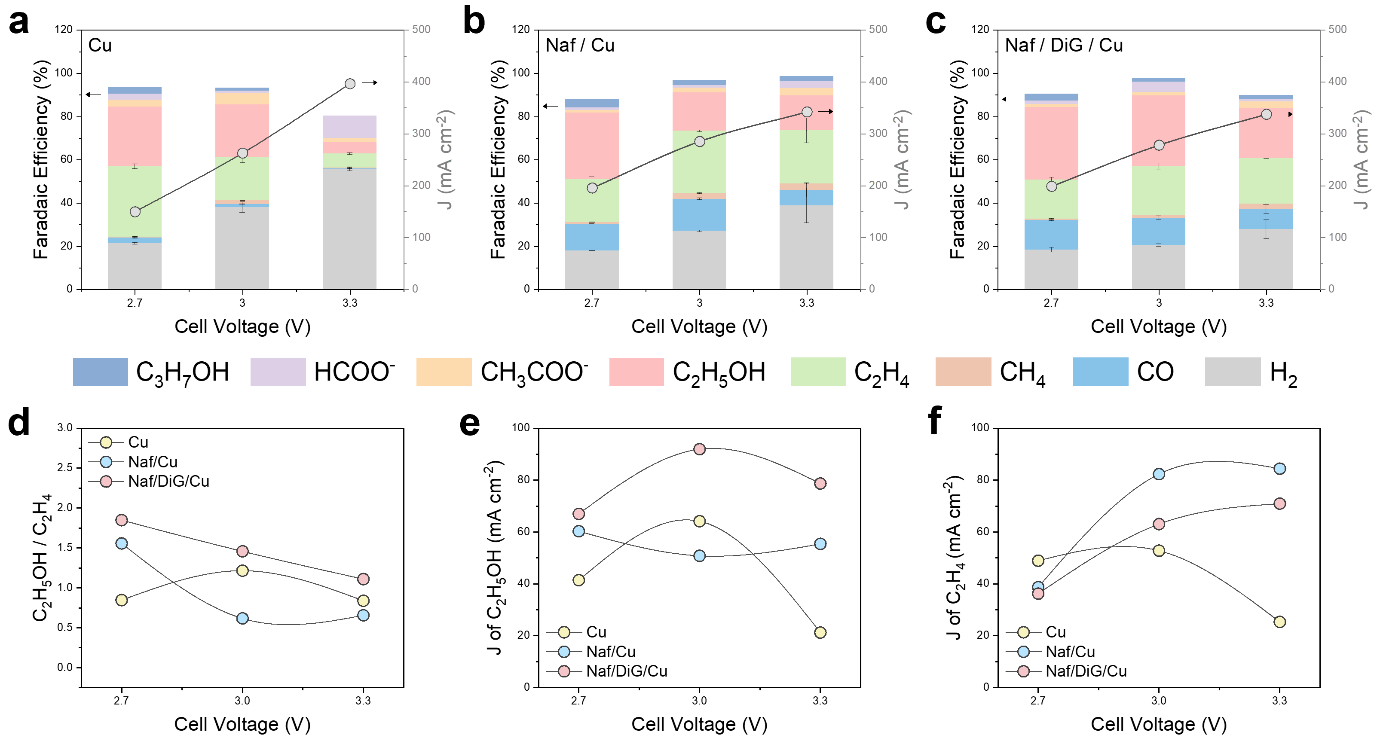


**Fig. S18 | CO_2_RR performance in 1 M KOH. a-c,** CO_2_RR product distribution of Cu (**a**), Naf/Cu (**b**), and Naf/DiG/Cu (**c**). **d**, Selectivity of C_2_H_5_OH compared to C_2_H_4_. **e-f**, Partial current density for C_2_H_5_OH (e) and C_2_H_4_ (**f**).


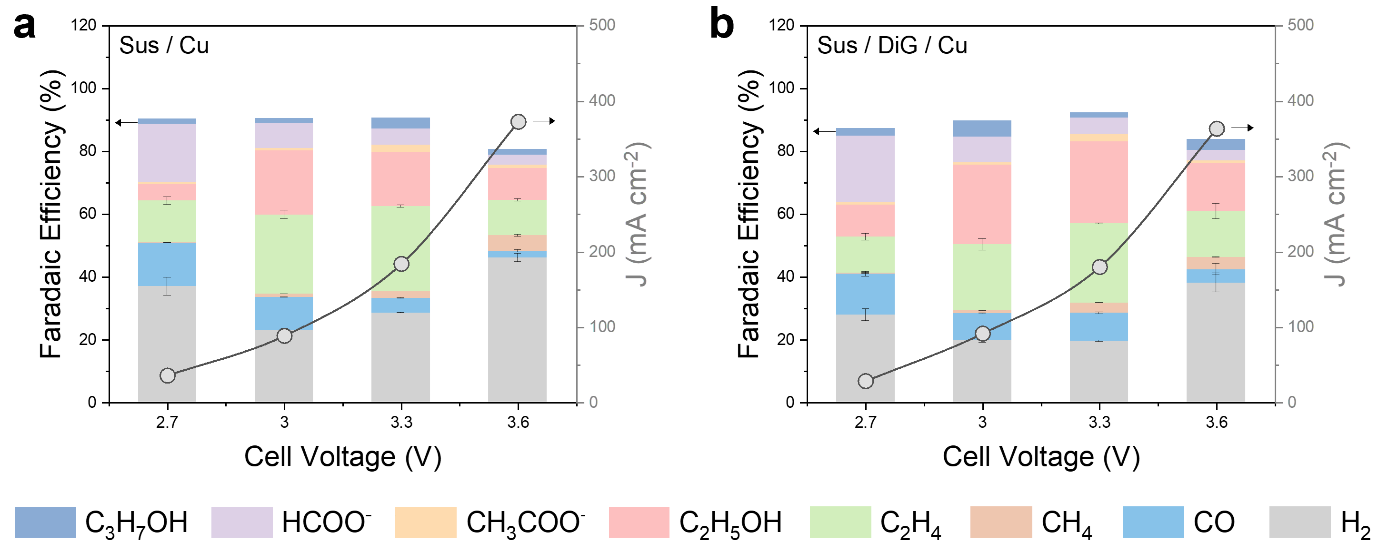


**Fig. S19 | Ionomer effect on CO_2_RR based on hetero-solvent incorporated Cu electrode. a-b,** CO_2_RR performance of Sus/Cu (**a**) and Sus/DiG/Cu (**b**).


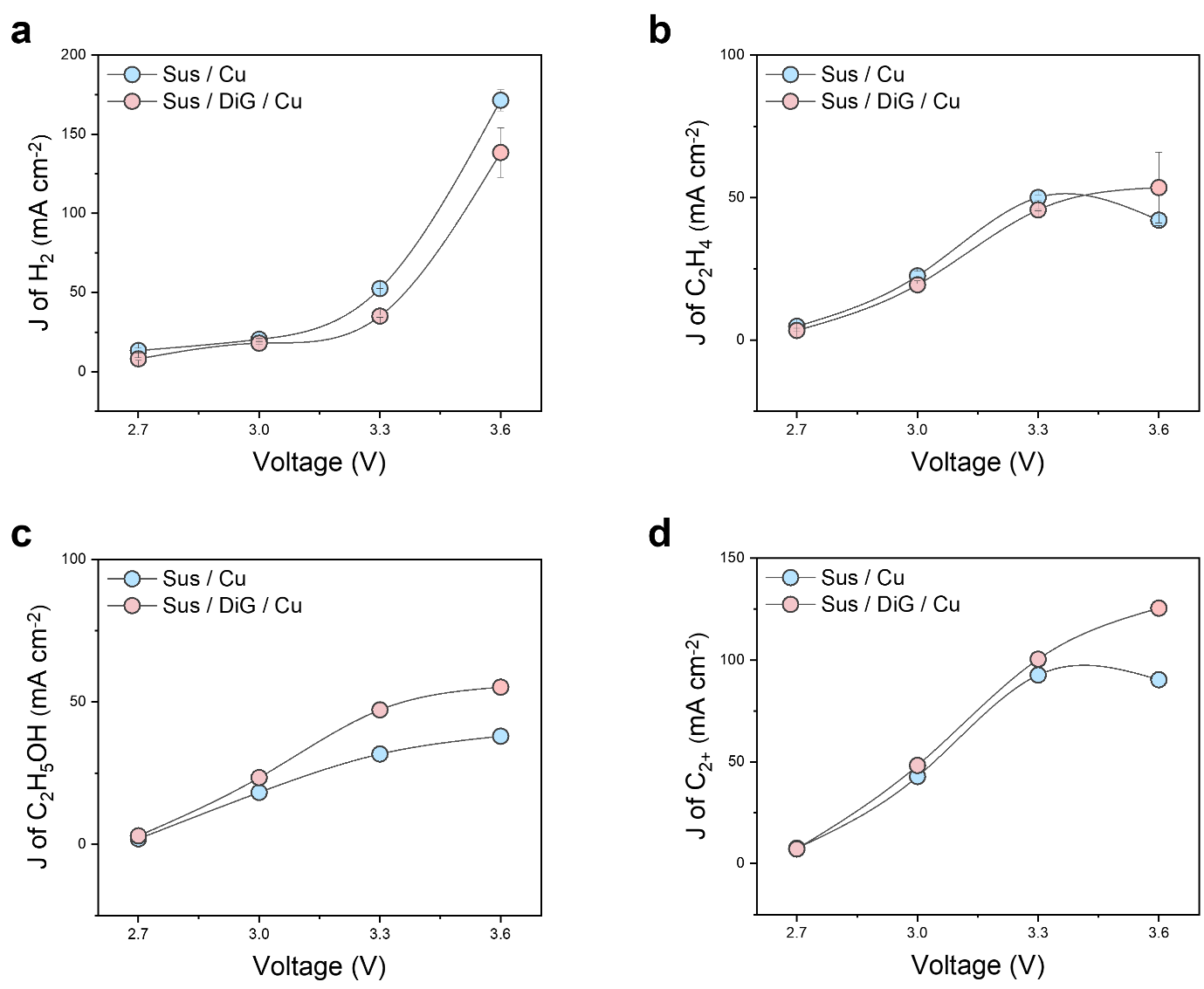


**Fig. S20 | Ionomer effect on CO_2_RR based on hetero-solvent incorporated Cu electrode. a-d,** Partial current density for H_2_ (**a**), C_2_H_4_ (**b**), C_2_H_5_OH (**c**), and C_2+_ (**d**).


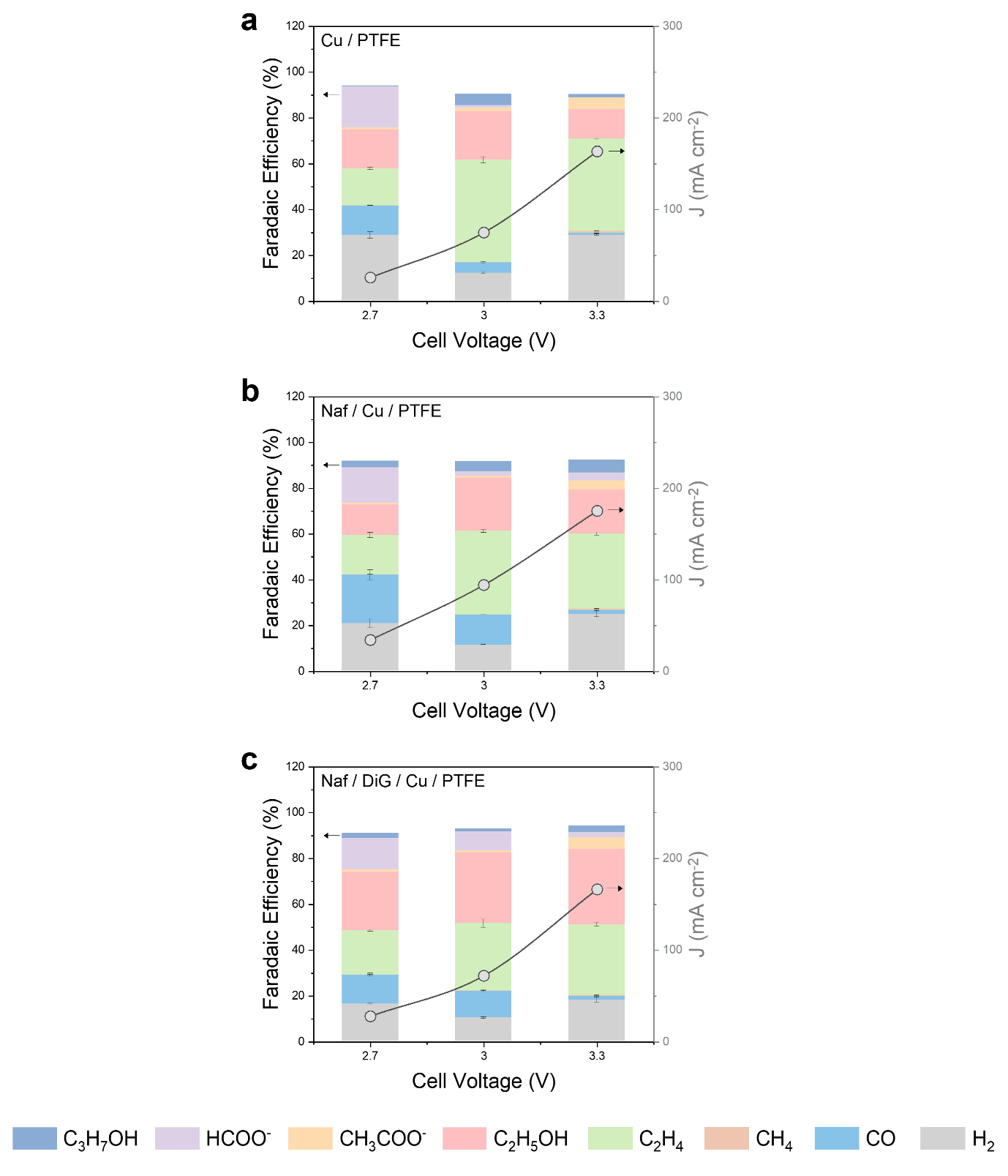


**Fig. S21** **| Substrate effect on CO_2_RR based on hetero-solvent incorporated Cu electrode. a-c,** CO_2_RR performance of Cu (**a**), Naf/Cu (**b**), and Naf/DiG/Cu (**c**) using PTFE substrate.


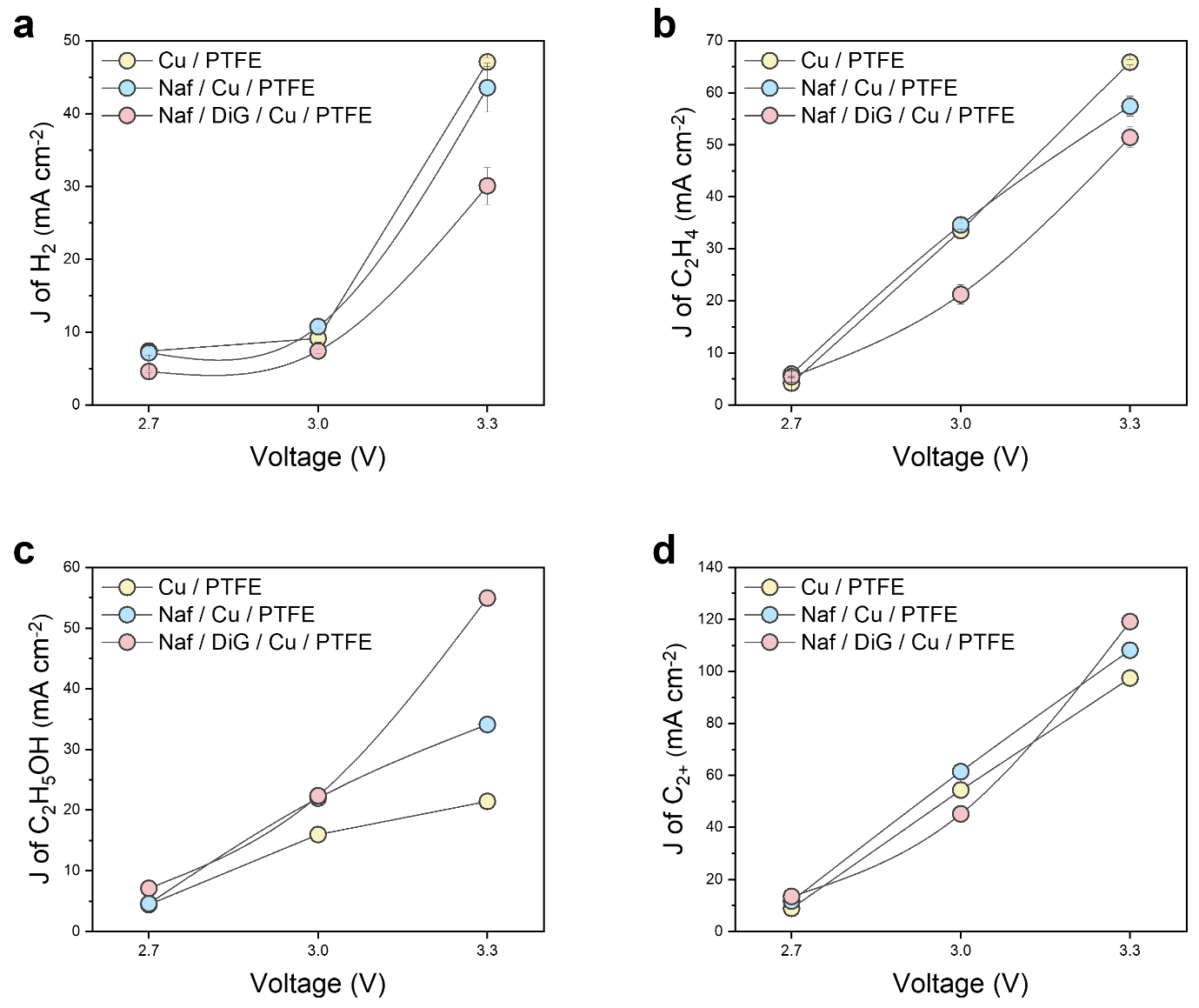


**Fig. S22 | Substrate effect on CO_2_RR based on hetero-solvent incorporated Cu electrode. a-d,** Partial current density for H_2_ (**a**), C_2_H_4_ (**b**), C_2_H_5_OH (**c**), and C_2+_ (**d**).


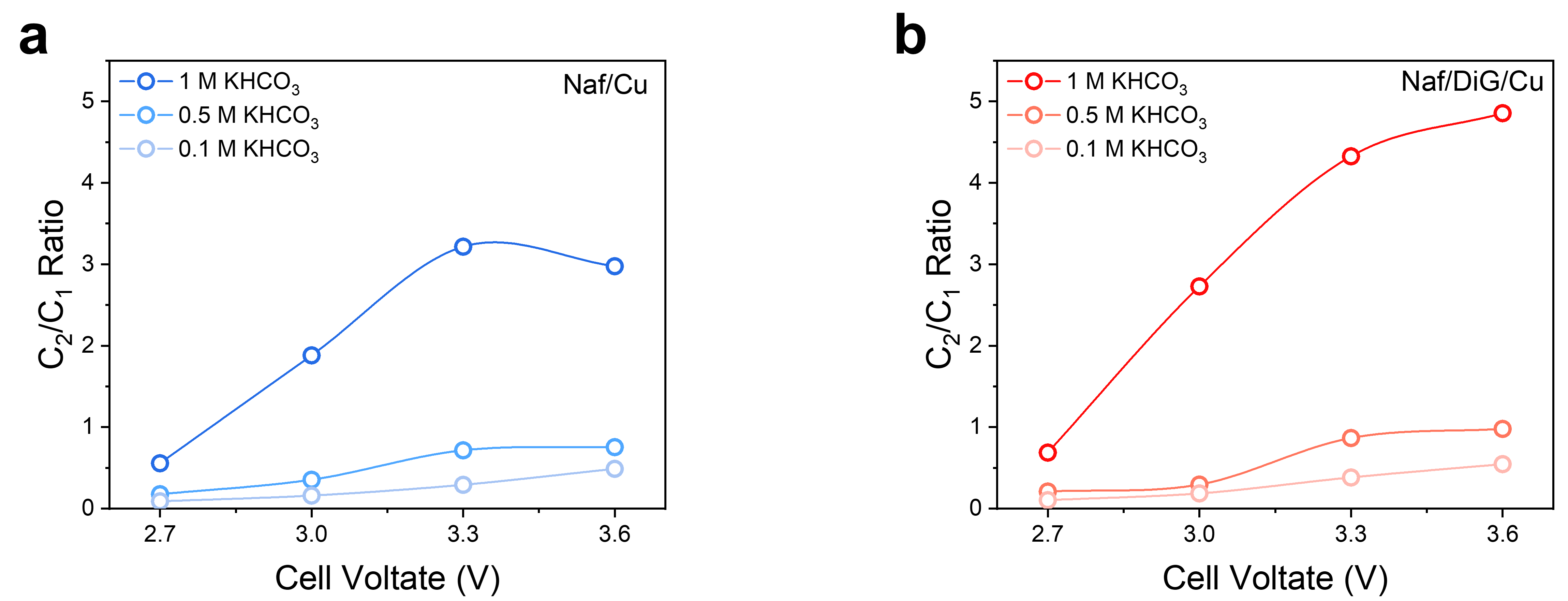


**Fig. S23 |** Selectivity for C_2_/C_1_ under various KHCO_3_ concentrations. C_2_/C_1_ ratio of (a) Naf/Cu and (b) Naf/DiG/Cu electrodes.


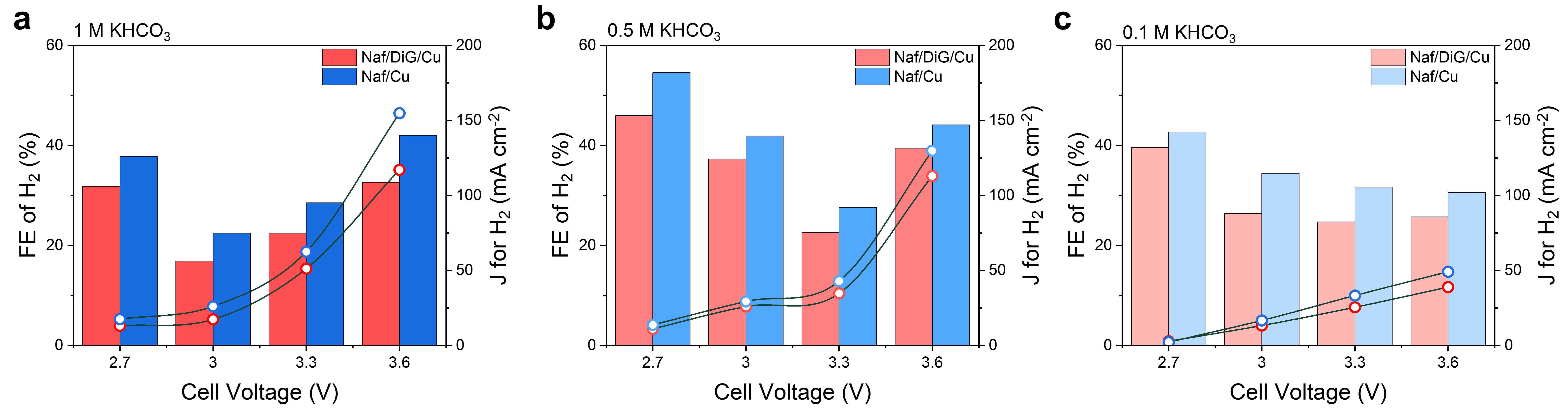


**Fig. S24 |** HER performance under various KHCO_3_ concentrations. FE and partial current densities for H_2_ at (a) 1 M, (b) 0.5 M, and (c) 0.1 M KHCO_3_.


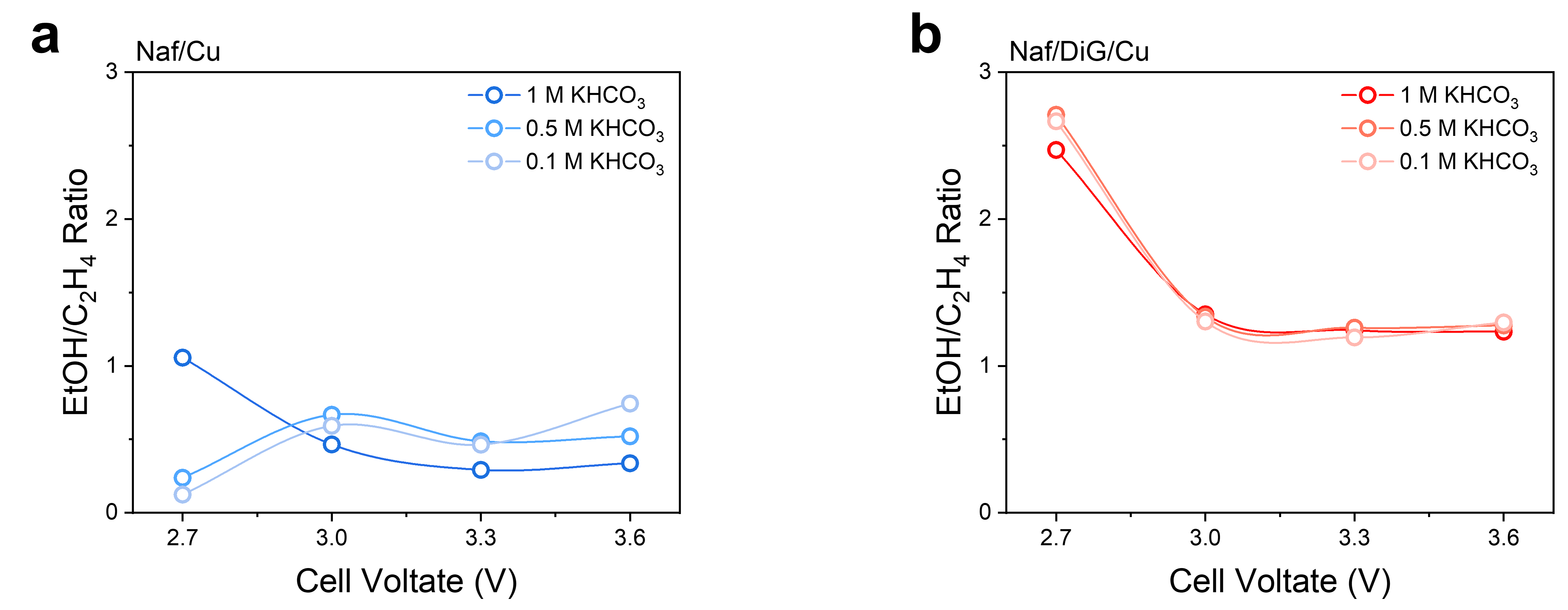


**Fig. S25 |** Selectivity for EtOH/C_2_H_4_ under various KHCO_3_ concentrations. EtOH/C_2_H_4_ ratio of (a) Naf/Cu and (b) Naf/DiG/Cu electrodes.


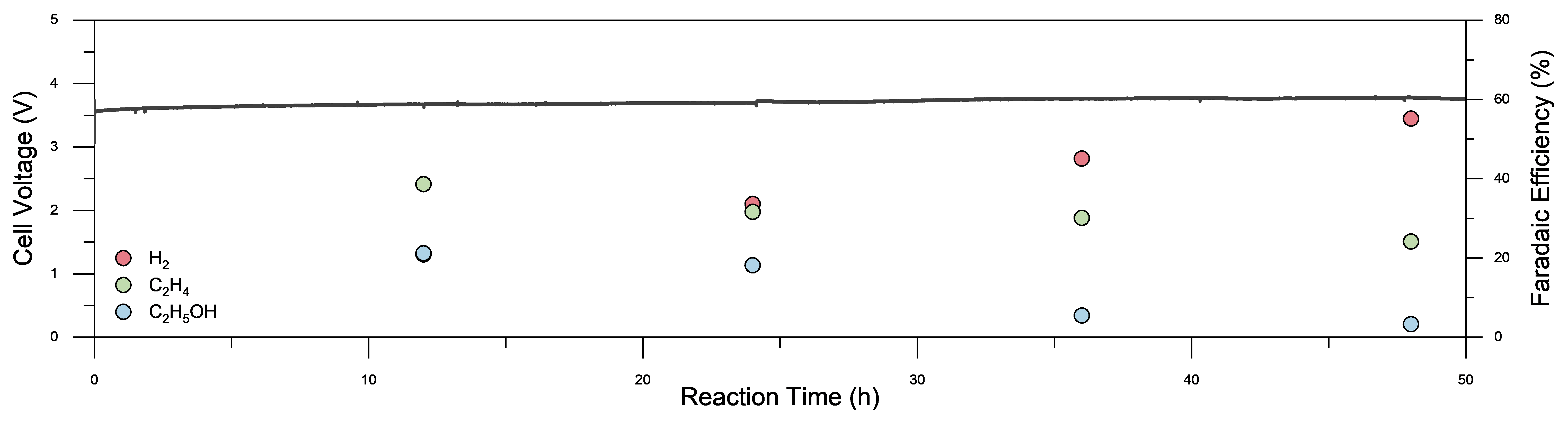


**Fig. S26 | Long-term stability measurement of Cu.** Cell voltage and FE of H_2_, C_2_H_4_, C_2_H_5_OH under 150 mA cm^-2^ during long-term CO_2_RR.


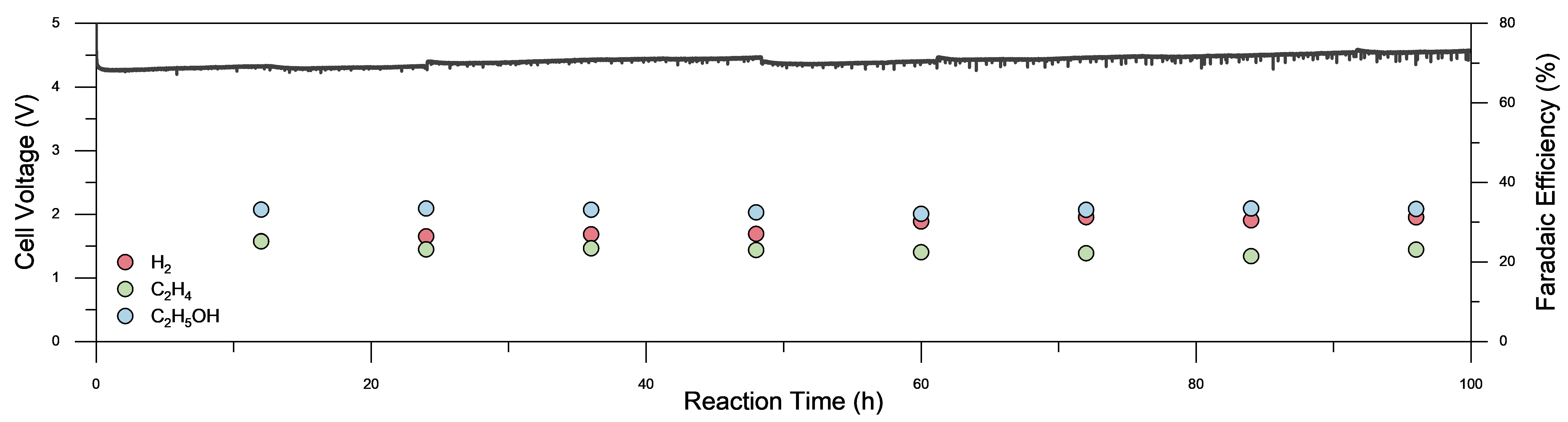


**Fig. S27 | Long-term stability measurement of Naf/DiG/Cu.** Cell voltage and FE of H_2_, C_2_H_4_, C_2_H_5_OH under 250 mA cm^-2^ during long-term CO_2_RR.


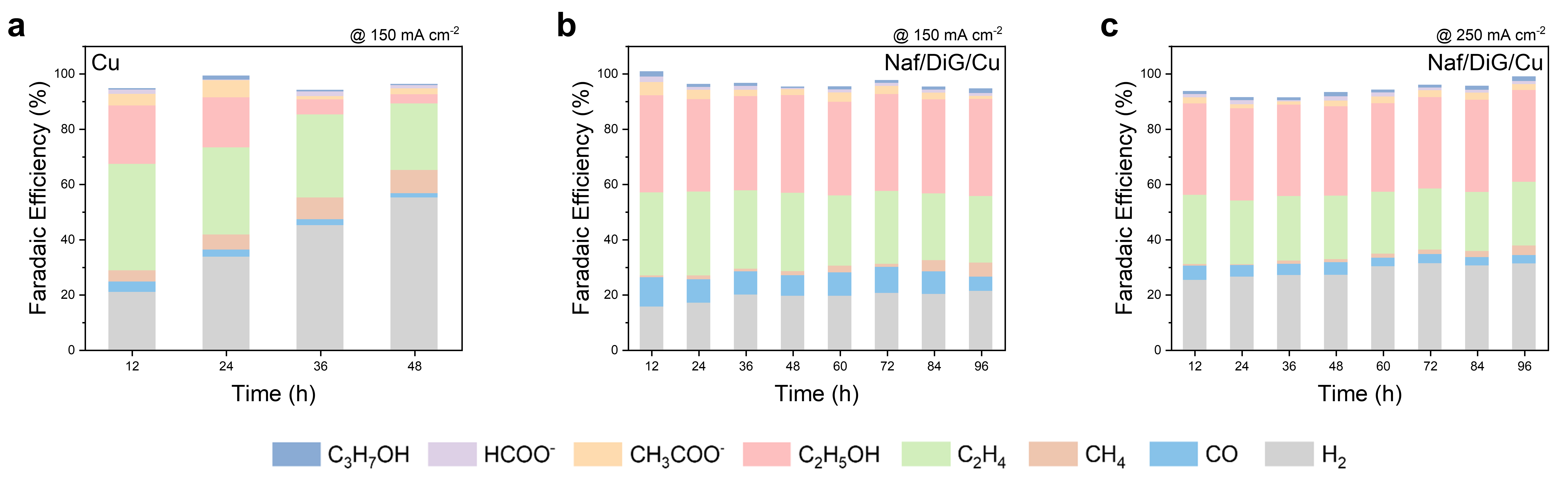


**Fig. S28 | CO_2_RR performance in long-term stability.** CO_2_RR average FE of Cu and Naf/DiG/Cu under 150 and 250 mA cm^-2^.


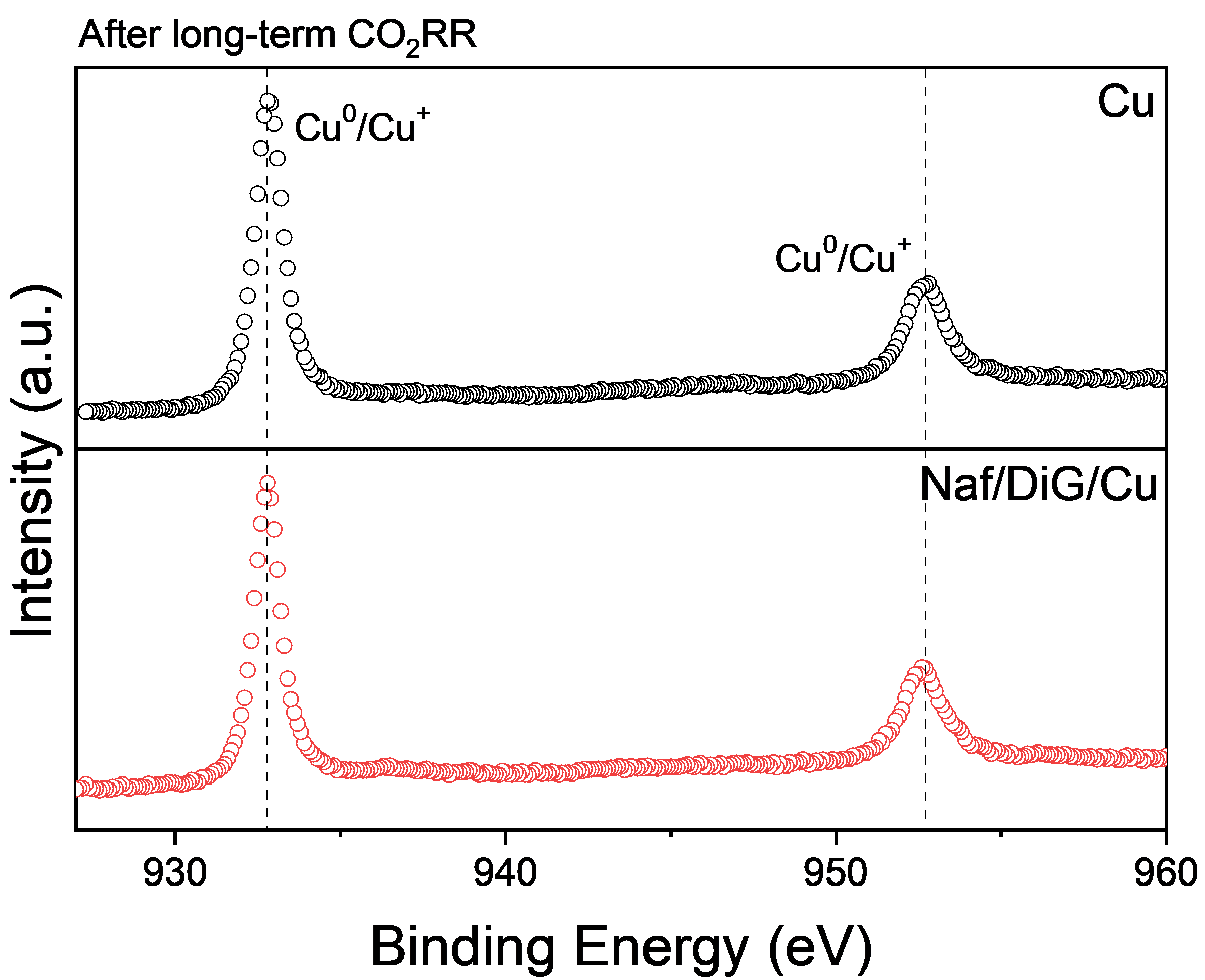


**Fig. S29 |** XPS spectra for Cu and Naf/DiG/Cu after long-term CO_2_RR

**
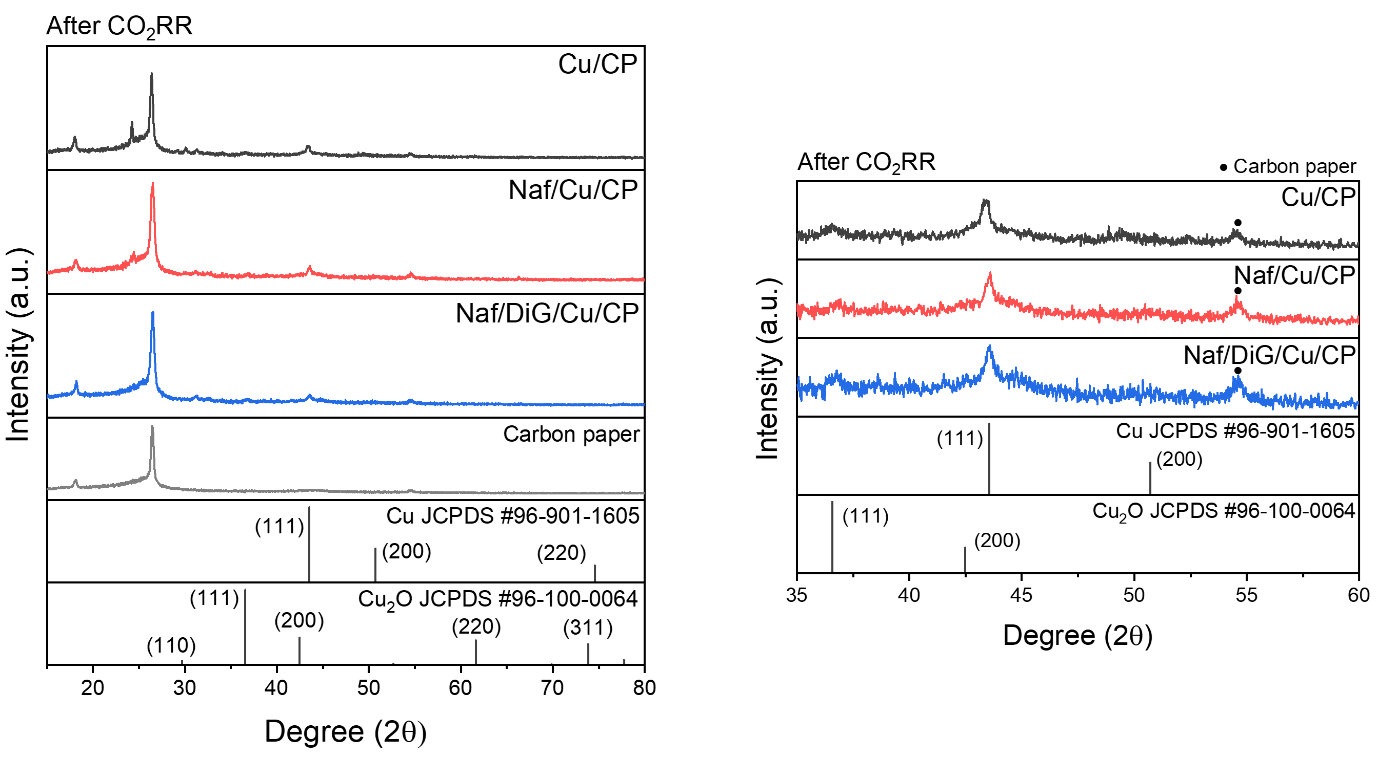
**

**Fig. S30 | XRD analysis after CO_2_RR.** XRD patterns of Cu, Naf/Cu, Naf/DiG/Cu on CP after CO_2_RR.


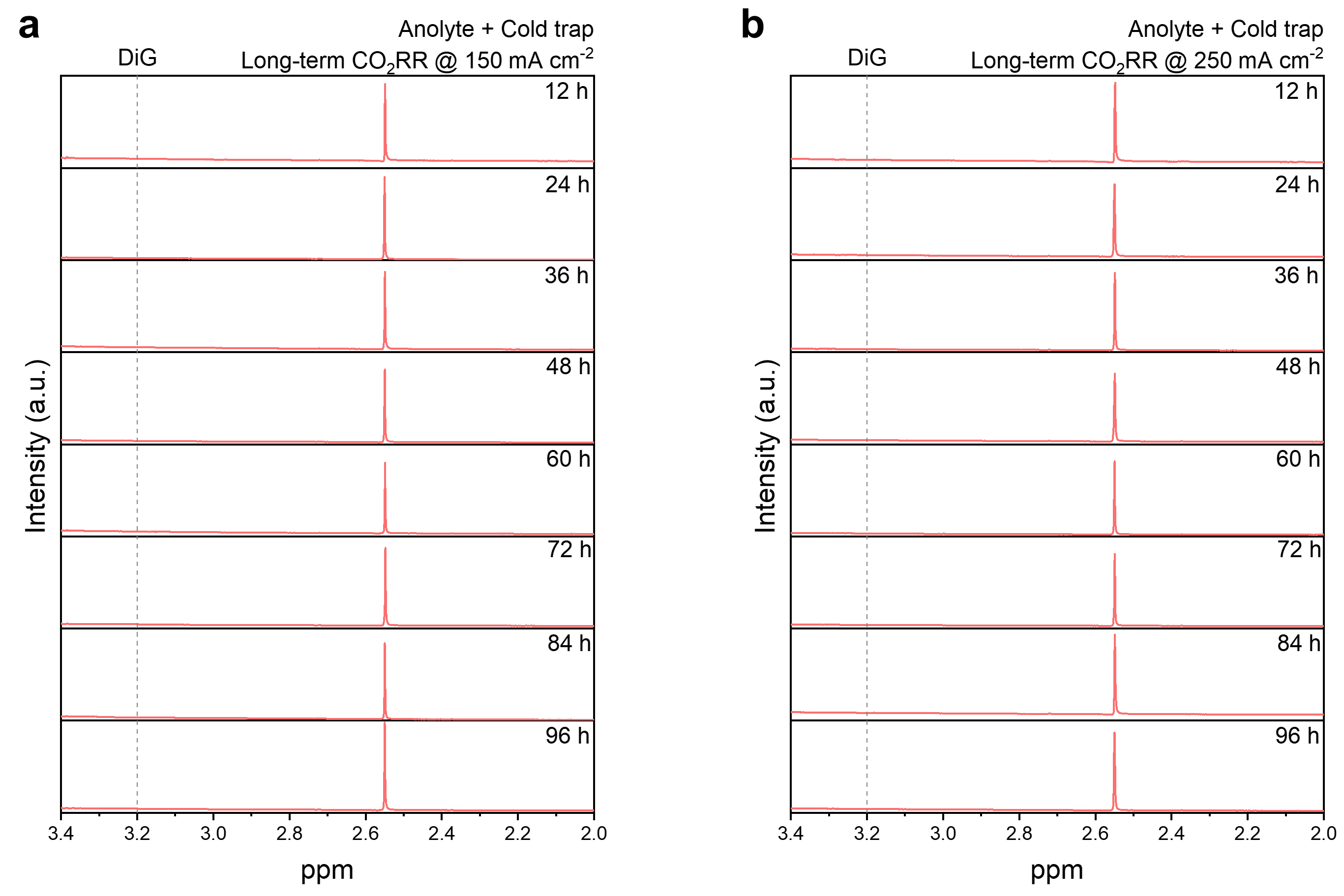


**Fig. S31 |** NMR spectra during long-term CO2RR of Naf/DiG/Cu under (a) 150 and (b) 250 mA cm^−2^.


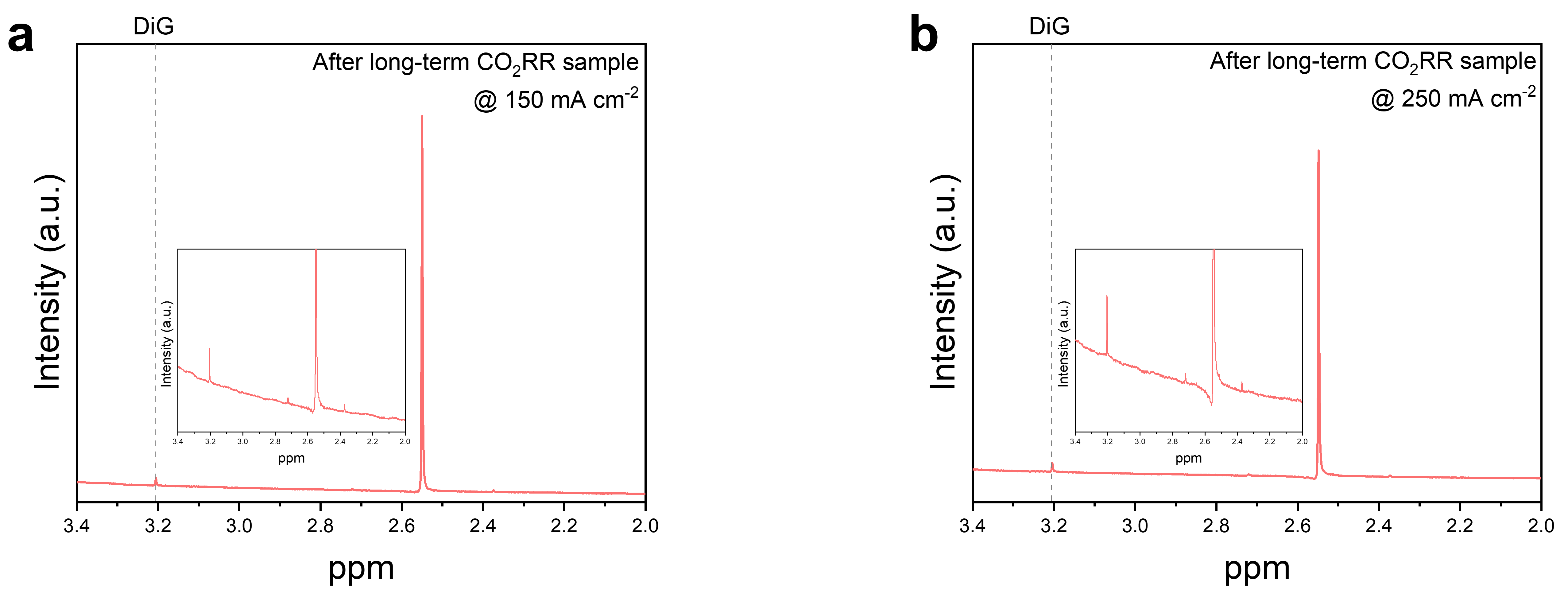


**Fig. S32 |** NMR spectra after long-term CO_2_RR for 100 h of membrane/Naf/DiG/Cu under (a) 150 and (b) 250 mA cm ^−2^.

**Table S2 |** State-of-the-art literature on ethanol production by MEA CO_2_RR in neutral media

| Catalysts | Anolyte | Cell voltage (V) | J_EtOH_  (mA cm^-2^) | EtOH/C_2_H_4_ | Strategy |
| --- | --- | --- | --- | --- | --- |
| Naf/DiG/Cu-Ag | 1 M KHCO_3_ | 3.6 | 184.2 | 2.6 | Microenvironment engineering (Hetero-solvent) |
| FeTPP[Cl]/Cu [S7] | 0.1 M KHCO_3_ | 3.7 | 49.2 | 1.1 | Molecule-metal interface engineering |
| Defect-rich Cu [S8] | 0.1 M KHCO_3_ | 3.5 | 95 | 0.5 | Defect engineering |
| Sputtered Cu [S9] | 0.1 M KHCO_3_ | 4.9 | 46 | - | Porous layer for ethanol separation |
| Porous Cu/QAPEEK [S10] | H_2_O | 3.54 | 65.7 | 0.1 | Bifunctional ionomer coating |
| Cu_2_OZn [S11] | H_2_O | 4.29 | 148.2 | 2.3 | Alloy |
| Cu-poly-1 [S12] | 1 M KHCO_3_ | 3.98 | 88.9 | 0.4 | Functionalized Cu NPs with fluoric polymer |

**Supplementary References**

1. M. Jödecke, Á. Pérez-Salado Kamps, G. Maurer, An experimental investigation of the solubility of CO_2_ in (N, N-dimethylmethanamide + water). J. Chem. Eng. Data **57**(4), 1249–1266 (2012). <https://doi.org/10.1021/je300105q>
2. X. Yin, J. Wang, S. Shen, Solubility of N_2_O and CO_2_ in dimethyl sulfoxide, 2-(butylamino)ethanol, and their water-lean blends. J. Chem. Eng. Data **69**(4), 1668–1676 (2024). <https://doi.org/10.1021/acs.jced.4c00015>
3. S. Joerg, R.S. Drago, J. Adams, Donor–acceptor and polarity parameters for hydrogen bonding solvents. J. Chem. Soc., Perkin Trans. 2 (11), 2431–2438 (1997). <https://doi.org/10.1039/a701041i>
4. R.J. Gomes, C. Birch, M.M. Cencer, C. Li, S.-B. Son et al., Probing electrolyte influence on CO_2_ reduction in aprotic solvents. J. Phys. Chem. C **126**(32), 13595–13606 (2022). <https://doi.org/10.1021/acs.jpcc.2c03321>
5. P.J. Linstrom, W.G. Mallard, The NIST chemistry WebBook:   a chemical data resource on the Internet. J. Chem. Eng. Data **46**(5), 1059–1063 (2001). <https://doi.org/10.1021/je000236i>
6. C. Liang, Y. Katayama, Y. Tao, A. Morinaga, B. Moss et al., Role of electrolyte pH on water oxidation for iridium oxides. J. Am. Chem. Soc. **146**(13), 8928–8938 (2024). <https://doi.org/10.1021/jacs.3c12011>
7. F. Li, Y.C. Li, Z. Wang, J. Li, D.-H. Nam et al., Cooperative CO_2_-to-ethanol conversion *via* enriched intermediates at molecule–metal catalyst interfaces. Nat. Catal. **3**(1), 75–82 (2020). <https://doi.org/10.1038/s41929-019-0383-7>
8. Z. Gu, H. Shen, Z. Chen, Y. Yang, C. Yang et al., Efficient electrocatalytic CO_2_ reduction to C^2+^ alcohols at defect-site-rich Cu surface. Joule **5**(2), 429–440 (2021). <https://doi.org/10.1016/j.joule.2020.12.011>
9. R.K. Miao, Y. Xu, A. Ozden, A. Robb, C.P. O’Brien et al., Electroosmotic flow steers neutral products and enables concentrated ethanol electroproduction from CO_2_. Joule **5**(10), 2742–2753 (2021). <https://doi.org/10.1016/j.joule.2021.08.013>
10. W. Li, Z. Yin, Z. Gao, G. Wang, Z. Li et al., Bifunctional ionomers for efficient co-electrolysis of CO_2_ and pure water towards ethylene production at industrial-scale current densities. Nat. Energy **7**(9), 835–843 (2022). <https://doi.org/10.1038/s41560-022-01092-9>
11. T.-U. Wi, R. University, Z.H. Levell, S. Hao et al., Selective and stable ethanol synthesis *via* electrochemical CO_2_ reduction in a solid electrolyte reactor. ACS Energy Lett. **10**(2), 822–829 (2025). <https://doi.org/10.1021/acsenergylett.4c03091>
12. T. Zhao, X. Zong, J. Liu, J. Chen, K. Xu et al., Functionalizing Cu nanoparticles with fluoric polymer to enhance C^2+^ product selectivity in membraned CO_2_ reduction. Appl. Catal. B Environ. **340**, 123281 (2024). <https://doi.org/10.1016/j.apcatb.2023.123281>
